# High-Precision Intrinsic Interactome Elucidation of Chimeric Antigen Receptors via Photocatalytic Micromapping (μMap-CAR)

**DOI:** 10.1021/jacs.6c08969

**Published:** 2026-07-01

**Authors:** Sean W. Huth, Chenmengxiao Roderick Pan, Philip Raftopoulos, Ciaran P. Seath, Gabrielle H. Lovett, Beryl Li, Sushma Yechan Gunja, Vaishali Agarwal, Yuka Amako, Harris Bell-Temin, Helen Pham, Helen Evans, Jennifer X. Qiao, Haibo Liu, Brook Barajas, David W.C. MacMillan

**Affiliations:** † 6740 Merck Center for Catalysis at Princeton University, Princeton, New Jersey 08544, United States; ‡ Department of Chemistry, Princeton University, Princeton, New Jersey 08544, United States; § 3971 Bristol Myers Squibb, Seattle, Washington 98109, United States; ∥ Discovery & Development Sciences, 3971 Bristol Myers Squibb, Cambridge, Massachusetts 02141, United States

## Abstract

Chimeric Antigen Receptor T-cell (CAR-T) therapies represent a powerful modality for treating a variety of hematological cancers. However, limited efficacy in broader disease contexts, particularly solid tumors, underscores the need for improved CAR design to enhance potency, persistence, and safety. Key design features of CARs have been profoundly informed by extensive knowledge of the T-cell receptor (TCR) and its interactome. To advance CAR-T therapies, new functionally relevant proteins are needed to support engineering efforts. Until now, the actual molecular microenvironment surrounding CARs has remained poorly defined, chiefly due to the lack of a characterization method with the required precision and sensitivity. Herein, we introduce μMap-CAR, a high-resolution photocatalytic proximity labeling platform featuring key methodological optimizations that enable direct elucidation of the CAR interactome on live T-cell surfaces. The platform performs robustly in a model CAR-T cell system under both resting and simulated activation conditions. The high sensitivity of μMap-CAR allows interrogation of interactome differences upon CAR endodomain alterations, linking perturbed signaling networks directly to CAR components. We further deliver the first high-resolution intrinsic CAR interactome in primary T-cells, defined by shared interactors across donors, and validate candidates via super-resolution microscopy and CAR-T activation perturbation. This platform constitutes a powerful, broadly applicable tool for CAR interactome profiling while also providing actionable targets for proximity-guided CAR engineering applications.

## Graphical abstract

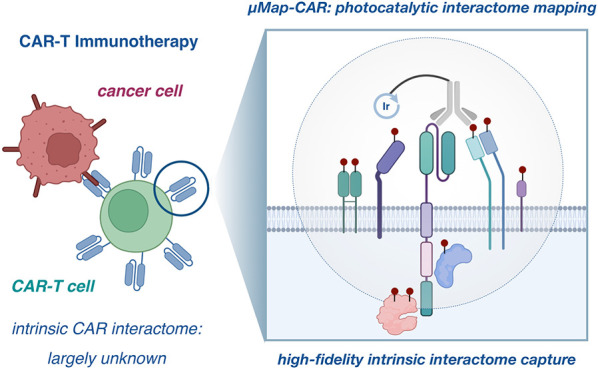

## 1 Introduction

Chimeric Antigen Receptor T-cell therapy (CAR-T) has emerged as a revolutionary immunotherapy for hematological cancers, with approved therapies targeting various forms of myeloma and lymphoma.^1–3^ A typical CAR construct consists of an extracellular antigen-binding domain (ScFv, Single-Chain Variable Fragment) directed against cancer-associated antigens such as CD19 or BCMA, a transmembrane linker domain (TM), one or more intracellular costimulatory domains, and the CD3ζ signaling chain (Figure 1A).^4–6^ When assembled, these domains recapitulate key aspects of native T-cell receptor (TCR) signaling, enabling engineered T cells to recognize and eliminate cancer cells.^7^

**Figure 1:**
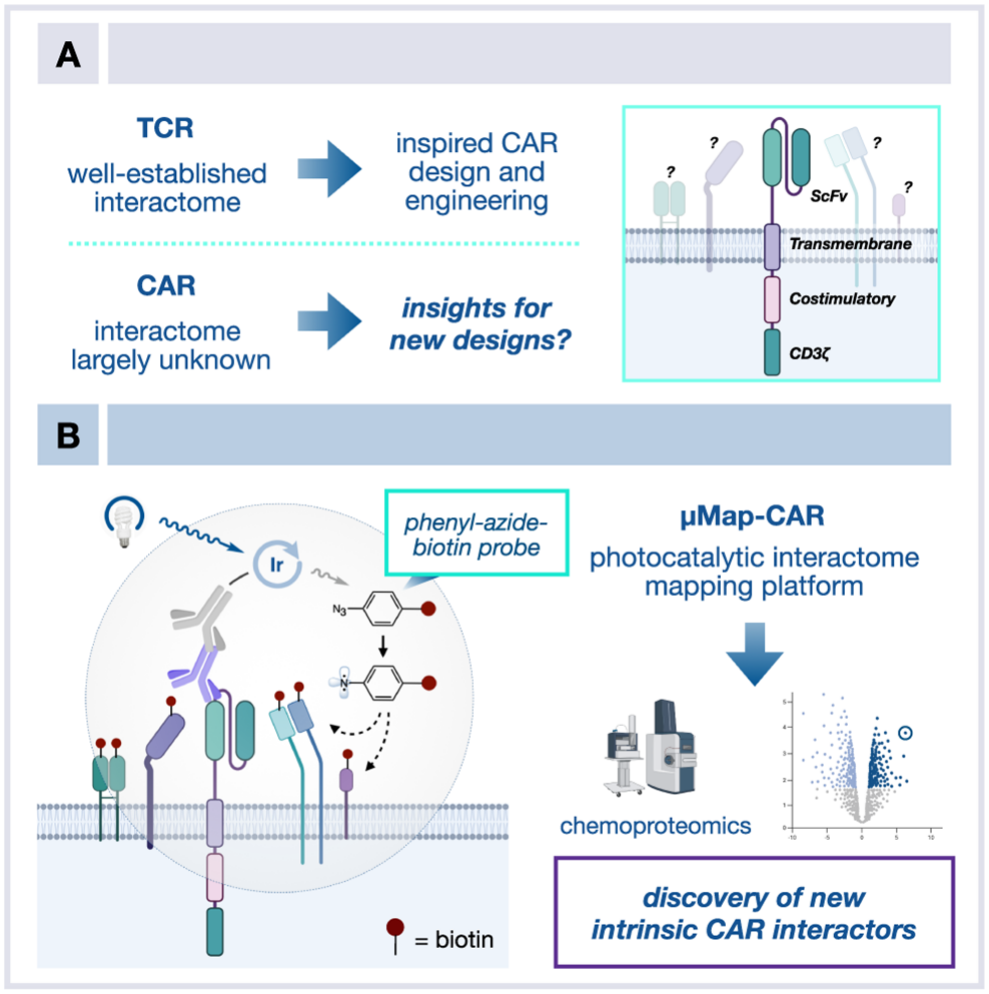
Overview of photocatalytic interactome mapping of chimeric antigen receptors for improved understanding of CAR-T biology. A. Hidden insights from the underexplored CAR interactome. B. This work: photocatalytic CAR interactome mapping.

Insights into the TCR microenvironment played a critical role in shaping early CAR design, driving the successful incorporation of CD28 and 4–1BB signaling motifs as costimulatory modules.^8^ However, emerging studies have revealed key differences between CAR and TCR immune synapses, including distinct spatial organization and signaling geometry, suggesting that TCR-derived models may not faithfully represent CAR biology.^9^ Moreover, although the function of core TCR signaling motifs is preserved in CAR, potential alterations in molecular interactions and regulatory networks within the chimeric CAR context have not been examined.^10,11^ Therefore, a detailed understanding of the CAR microenvironment is critical to inform next-generation CAR engineering, yet such knowledge remains limited because of the lack of biochemical tools with high spatial precision and sensitivity.^12,13^ While recent gene-editing-based screening approaches offer important insights into global CAR-T cell fitness, they fail to directly resolve the impact of specific CAR designs on the immediate molecular microenvironment and functional interactions, limiting the rational design of engineered proteins.^14–17^ A platform for comprehensive profiling of the CAR interactome holds great promise in deepening our understanding of CAR function and inspiring next-generation designs aimed at overcoming limited efficacy, minimizing toxicity, and improving response rates.^18,19^

Herein, we introduce μMap-CAR, a high-resolution photocatalytic interactome-mapping platform that enables selective and sensitive profiling of the CAR microenvironment. Adapted from our high-resolution μMap photoproximity labeling platform,^20–23^ μMap-CAR localizes an iridium (Ir) photocatalyst to the CAR via a targeting antibody conjugate. Phenyl-azide probes are then photocatalytically activated under blue-light irradiation to covalently append biotin handles to neighboring proteins (within ∼120 nm), enabling downstream enrichment and chemoproteomic analysis (Figure 1B).^24,25^ μMap-CAR provides sufficient spatial resolution and sensitivity to reveal perturbation-induced changes in CAR-associated interaction networks and capture the intrinsic CAR microenvironment in primary CAR-T cells across multiple donors. By cross-referencing these data sets, we identify previously unrecognized CAR-proximal proteins with potential roles in regulating CAR signaling and T-cell function, thereby enabling proximity-informed strategies for engineering next-generation CAR-T therapies.

## 2 Results

### 2.1 Development of μMap-CAR for Activation-Dependent CAR Interactome Mapping

To establish proof of concept for our labeling workflow, we used a Jurkat T-cell line expressing a second-generation anti-CD19 CAR with a 4–1BB costimulatory domain. This construct also contains an HA tag on the CAR extracellular domain for antibody targeting (Figure 2A). Upon binding of the HA antibody to the CAR at 37 °C, reporter cell line assays demonstrated a measurable increase in both Nur77 gene expression, which represents CD3ζ signaling, and NFκB pathway activation, which also reflects active costimulation (Figure 2B).^26,27^ This indicated that an activated CAR state could be mimicked via incubation of the HA antibody at physiological temperatures, while activity was significantly suppressed at 4 °C, allowing for the capture of activation-dependent CAR interactors.

**Figure 2:**
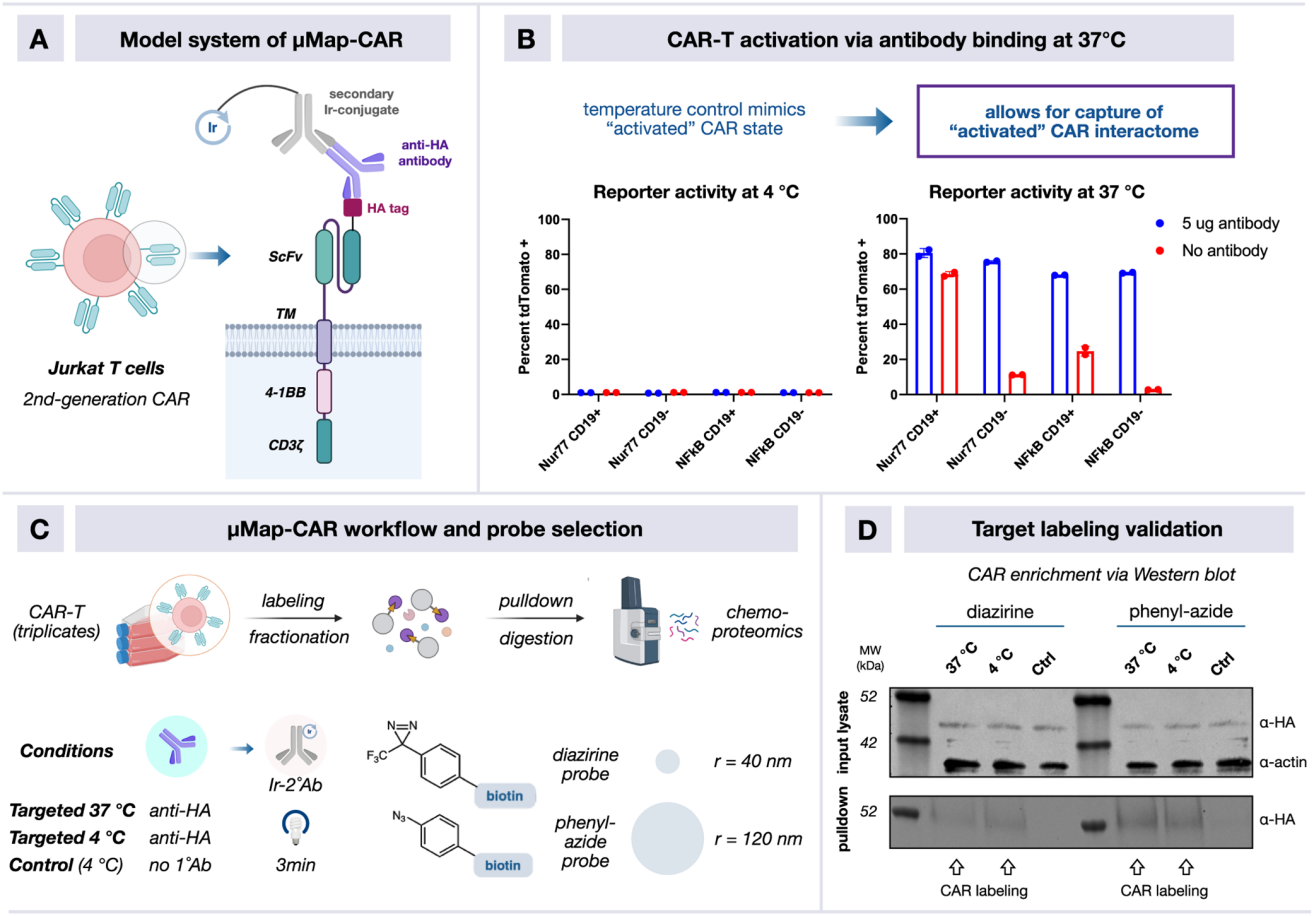
(a) General μMap-CAR labeling schematic, showing the antibody–catalyst conjugate binding to an HA tag on the extracellular domain of the CAR. (b) Reporter cell lines demonstrate CAR signaling upon HA antibody binding at 37 °C for both NFκB and Nur77 pathways. Two biological replicates were subjected to flow cytometry analysis. (c) Workflow of μMap-CAR. Key labeling conditions are indicated, including variations in temperature, primary antibody treatment, and photocatalytic probe. For proteomic analysis, CAR-T samples were prepared in triplicate for each condition. (d) Western blot shows enrichment of biotinylated HA-CAR in the streptavidin pulldown fraction of the labeled samples. Results were validated via independent repeats.

Encouraged by these reporter results, we next sought to confirm CAR labeling via the μMap workflow (Figure 2C). CAR-T Jurkat cells were subjected to either primary HA antibody (targeted, at 37 °C or 4 °C) or no primary incubation (control, 4 °C). For samples activated at 37 °C, cells were rapidly cooled to 4 °C after primary incubation as part of an activation-locking workflow to preserve the surface CAR interactome. All subsequent steps were then carried out at 4 °C, including incubation with the secondary Ir-conjugated antibody, diazirine-biotin or phenyl-azide-biotin, and 3 min of blue light irradiation to finish labeling. Subsequent lysis and pulldown revealed CAR enrichment by Western blot using both photoactivated probes in only the targeted arms (Figure 2D). Having validated CAR labeling, we performed μMap-CAR with a downstream chemoproteomics workflow to profile the CAR interactome, using triplicate samples for each condition.

Notably, CAR was the top-enriched hit in the targeted arms using both probes (Figure 3A). We further analyzed highly enriched proteins (Log_2_(Fold Change)>1, −Log­(P-value)>1.3) to identify candidates likely to interact directly, for instance, with individual CAR endodomains. To this end, we anticipated that the 4–1BB domain would recruit TRAF proteins, adaptors crucial to initiating signaling pathways,^28^ while the CD3ζ domain would be expected to recruit ZAP70, a critical kinase in the phosphorylation signaling cascade during T cell activation.^29^ The diazirine probe yielded a limited number of highly enriched proximal proteins, which may reflect both the restricted spatial reach of the activated carbene radicals and the relatively few proteins in physical contact with CAR in the microclusters during signaling activation (Figure 3A).^30,31^ In comparison, the phenyl-azide probe, which offers a modestly expanded labeling radius, captured a broader group of candidates, among which both TRAF and ZAP70 were identified as top interactors, indicating successful profiling of the CAR interactome in our model system (Figure 3A). These results underscore the value of tunable labeling radii of μMap in resolving distinct layers of the surface interactome.^25^ Further analysis of the targeted arm revealed enrichment of a variety of known TCR pathway components, including CD3ζ-associated signaling proteins and immune regulators. Additional gene ontology (GO) and STRING analysis highlighted proteins involved in adhesion and cellular import machinery (Figure S1).

**Figure 3:**
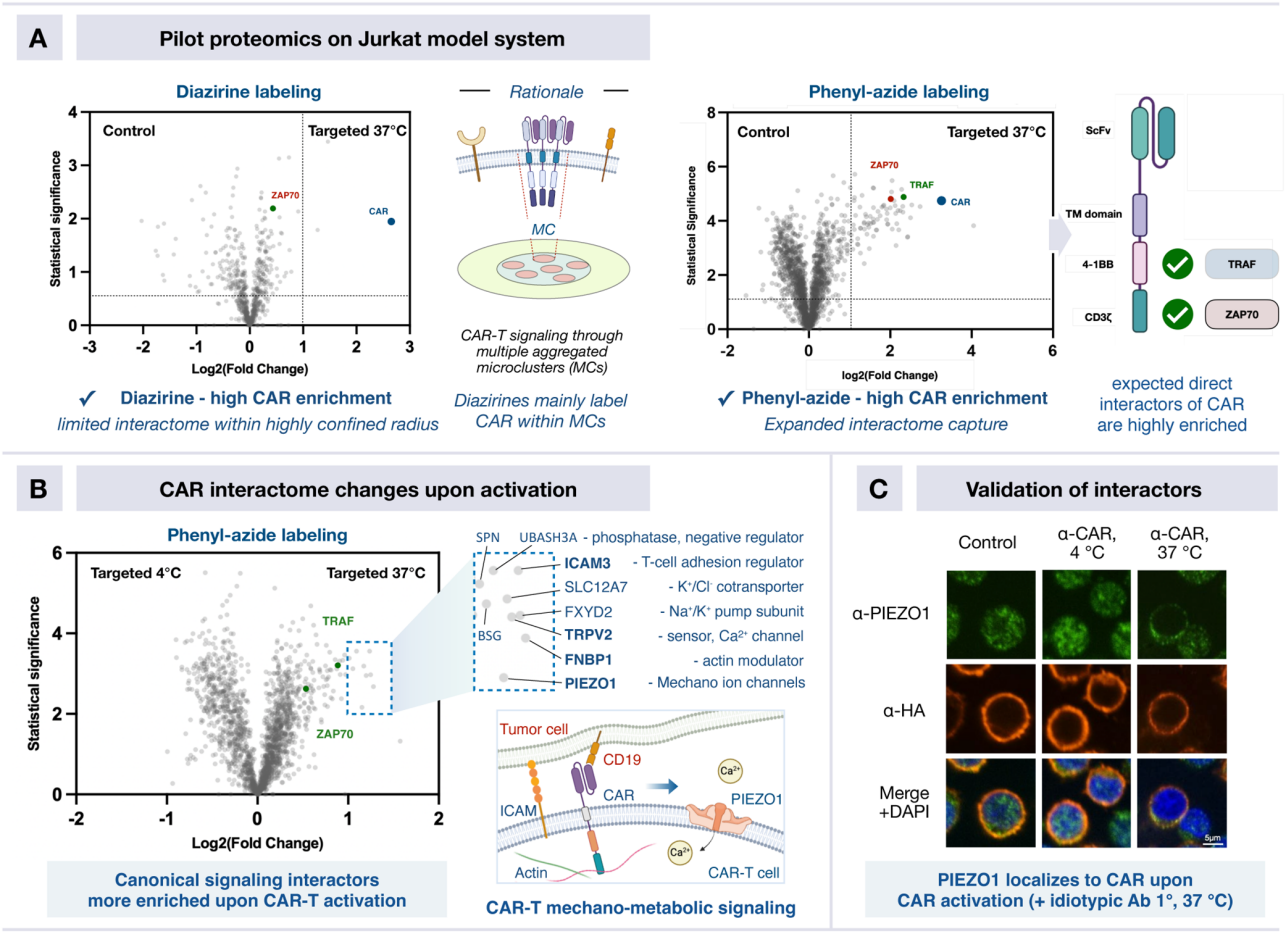
(a) Proof-of-concept volcano plots demonstrate high enrichment of CAR from both probes (targeted 37 °C to control, two-tailed Student’s *t*-test, triplicate in each condition). Direct binding interactors of 4–1BB (TRAF), as well as CD3ζ (ZAP70), are highly enriched using the phenyl-azide probe (Log_2_(Fold Change)>1, −Log­(P-value)>1.3). (b) Activation-associated changes in the CAR interactome were revealed by comparing targeted 37 °C and targeted 4 °C (two-tailed Student’s *t*-test, triplicate in each condition). Annotated hits (Log_2_(Fold Change)>1, −Log­(P-value)>1.3) suggest the functional relevance of the CAR-T mechano-metabolic signaling pathway, with key interactors highlighted in the schematic. (c) Immunofluorescence observation of PIEZO1 localizing to the membrane CAR upon activation with anti-CAR idiotypic antibody at 37 °C. Results were validated via independent repeats.

On the basis of these results, we selected the larger-radius phenyl-azide probe for subsequent analysis. We compared the resting and activated state CAR interactomes to identify activation-associated changes that might reflect functionally relevant recruitment (Figure 3B). Many interactors showed increased active-state enrichment, including TRAF and ZAP70, consistent with their roles in CAR signaling. Notably, the data set also highlighted molecules implicated in mechanometabolic signaling and cytoskeletal dynamics, such as adhesion-related proteins (e.g., ICAM3), mechanosensitive ion channels (including PIEZO1 and TRPV2), and regulators of actin remodeling (e.g., FNBP1). These candidates align with emerging evidence that mechanosensitive channels, such as PIEZO1, couple mechanical forces at the immune synapse to metabolic and signaling pathways that influence T cell activation and effector function.^32,33^ Although CAR activation in this model was induced using target antibodies as surrogate stimuli rather than antigen-expressing tumor cells, the enrichment of mechanometabolic and cytoskeletal regulators suggests that key features of receptor activation are nonetheless captured. To validate one such candidate, PIEZO1, we performed immunofluorescence in CAR-expressing Jurkat T cells and observed activation-dependent colocalization with the CAR (Figure 3C), supporting recent work on its functional relevance in CAR-T biology.^34^

### 2.2 CAR Endodomain Mutations Rewire Local Interaction Networks Revealed by μMap-CAR

Engineering CAR endodomains is a key strategy for enhancing CAR-T therapies, often requiring precise modulation of signaling to optimize T-cell activation, survival, and persistence.^35,36^ To this end, we aimed to validate the sensitivity of μMap-CAR to interactome changes resulting from signal-altering modifications to cytosolic modules. Specifically, we examined three “loss-of-function” CAR mutants (Figure 4A): (1) CD3ζ 3× ITAM Mutant, a CAR with all three ITAMs (immunoreceptor tyrosine-based activation motifs) mutated in the CD3ζ region, leading to a loss of CD3-mediated ZAP70 binding and phosphorylation signaling (Figure S2);^37^ (2) 4–1BB 1× A Mutant: a CAR with a single amino acid mutation in both 4–1BB TRAF-interaction domains, which partially reduces TRAF recruitment capacity; and (3) 4–1BB 2× A Mutant: a CAR containing two mutations in both 4–1BB TRAF-interaction domains that should completely ablate this interaction.^38^

**Figure 4:**
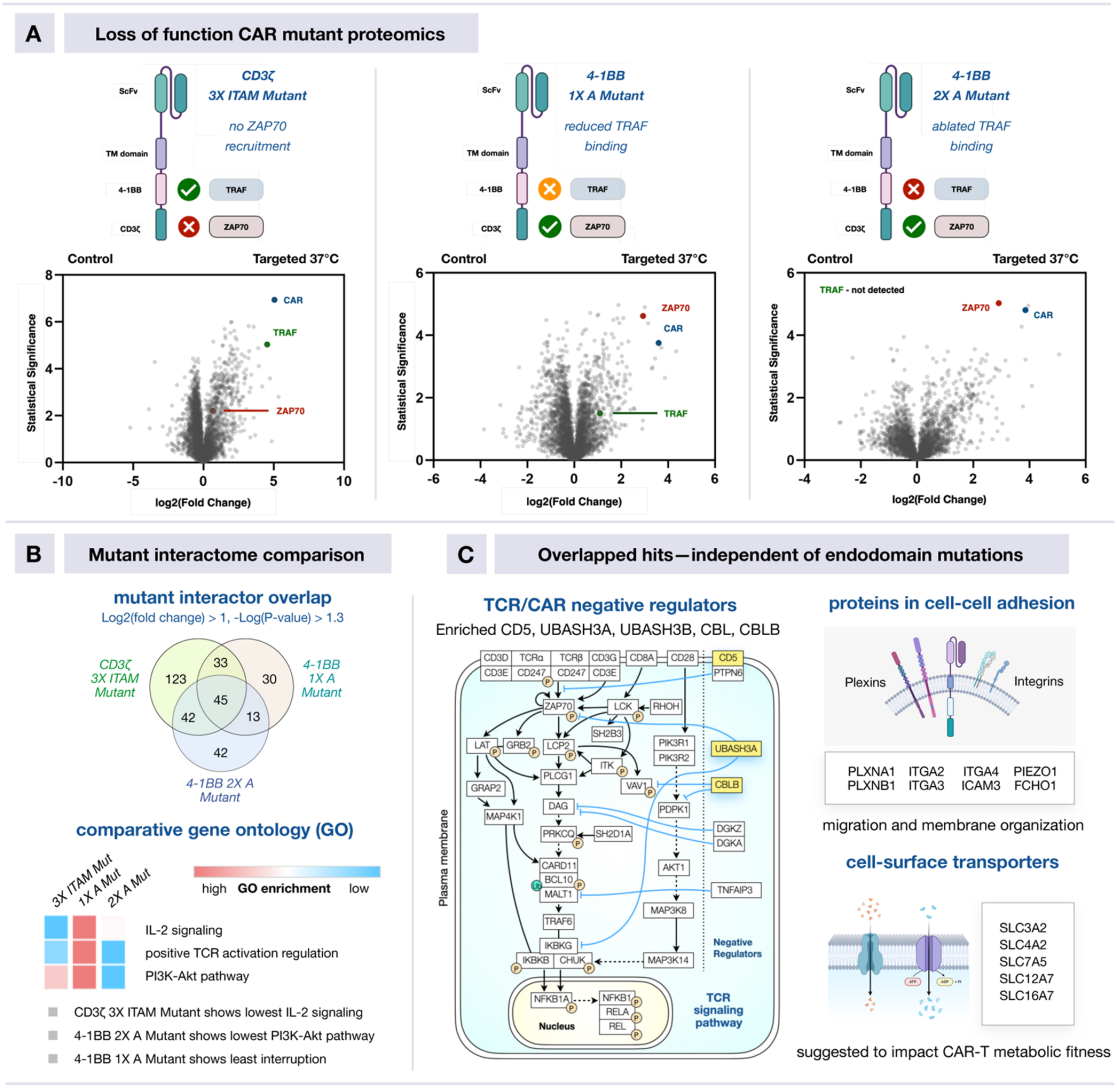
(a) Proteomics performed on CAR constructs with loss-of-function mutations: 1) CD3ζ 3× ITAM mutant with ablated CD3 signaling; 2) 4–1BB 1× A mutant with reduced TRAF recruitment; 3) 4–1BB 2× A mutant with ablated TRAF recruitment (two-tailed Student’s *t*-test, triplicate in each condition). Proteomic enrichment of ZAP70/TRAF is consistent with signaling disruption. (b) Comparison of the interactome across mutants. A Venn diagram shows mutant interactome overlap and differences (Log_2_(Fold Change)>1, −Log­(P-value)>1.3). Comparative gene ontology shows reduced IL-2 signaling, TCR activation, and PI3K signaling in the CD3ζ 3× ITAM mutant and 4–1BB 2× A mutant compared to the 4–1BB 1× A mutant. (c) Major pathway and functional categories analysis of shared hits across mutants (*n* = 45) identifies TCR/CAR negative regulators, cell adhesion signaling, and cell-surface transporters as conserved features of the CAR interactome independent of TCR or costimulatory signaling domain perturbation.

Upon application of μMap-CAR to these modified constructs, we observed trends consistent with the corresponding loss of function (Figure 4A). The CD3ζ 3× ITAM mutant showed a notable loss of ZAP70 enrichment, consistent with inhibition of signaling following ITAM phosphorylation. As expected, high TRAF enrichment was observed in this experiment, revealing that the nearby 4–1BB domain, which was unaltered, still recruited TRAF. The 4–1BB 1× A mutant showed the opposite pattern of enrichment: ZAP70 was highly enriched, while TRAF showed much lower enrichment and was no longer among the top hits. Finally, in the 4–1BB 2× A mutant, TRAF was absent while ZAP70 remained highly enriched, demonstrating that the CD3ζ-ZAP70 interaction remains intact. Global proteomics revealed no major differences in the protein levels of key signaling proteins, such as ZAP70 and TRAF proteins, between cells expressing unmodified and 4–1BB 2× A mutant CARs, indicating that the observed enrichment changes reflect altered recruitment to the CAR microenvironment rather than expression levels (Figure S3).

Subsequent overlapping analysis and comparative GO analysis of these loss-of-function mutants revealed differential enrichment of certain signaling pathways, potentially indicating the pathways associated with each domain (Figure 4B, Figure S4). Specifically, IL-2 signaling was most diminished in the 3× ITAM Mutant, consistent with its dependence on canonical ZAP70-CD3 signaling, whose impairment is reflected by reduced enrichment of ZAP70-GRB2/CRKL axis compared with the wildtype CAR interactome (Figure S5).^39^ By contrast, the TRAF5-BIRC2/3 axis is no longer enriched in the 4–1BB 2× A Mutant, consistent with the complete ablation of costimulatory 4–1BB–TRAF signaling, which promotes Akt-dependent pro-survival pathways (Figure S6).^40,41^ Unsurprisingly, the 4–1BB 1× A Mutant, a hypomorph, showed the least disruption of downstream signaling among the three mutants.

Notably, a set of interactors (Figure 4B, n = 45) was enriched across multiple mutants, with around half of the proteins robustly captured in the wildtype CAR interactome, implying that their presence in the CAR microenvironment is independent of canonical CD3 or costimulatory signaling perturbation (Figure S7). These include novel interactors PIEZO1 and SLC12A7, along with well-defined TCR/CAR negative regulators, such as ZAP70 phosphatase UBASH3A/B, E3 ubiquitin ligase CBL/CBLB, and cell surface glycoprotein CD5 (Figure 4C).^42–44^ STRING analysis highlighted enrichment of proteins involved in the regulation of cell adhesion, including IGTAs, ICAM3, and PLXNs, which act to coordinate immune synapse formation and cytoskeletal organization to support sustained TCR/CAR signaling (Figure 4C).^45,46^ Surface transporters were also enriched, including SLC3A2-SLC7A5, the crucial heterodimeric amino acid transporter pair, suggesting their fundamental role in sustaining CAR-T metabolic fitness (Figure 4C). Together, these data provide the first example of domain-perturbed CAR interactome profiling, revealing both domain-specific and domain-independent interactions within the CAR signaling network and the surrounding regulatory landscape.

### 2.3 Cross-Donor Profiling Defines a High-Fidelity Intrinsic CAR Interactome in Primary T Cells

Having demonstrated that μMap-CAR can capture sensitive signaling changes in engineered CARs, we next aimed to translate the platform to primary human CAR-T cells. While the Jurkat model system provides a robust, scalable, and well-controlled platform for validating μMap-CAR and benchmarking CAR perturbations, primary human T cells more closely reflect the therapeutic context in which CARs operate. Extending our analysis to primary CAR-T cells enables the evaluation of CAR microenvironments under more biologically relevant conditions while preserving insights gained from the model system. To minimize perturbation of the native sequence and structure of the therapeutic anti-CD19 CAR, we eliminated the extracellular HA tag and instead utilized an anti-CAR idiotypic antibody to preserve CAR targeting (Figure 5A).^47^

**Figure 5:**
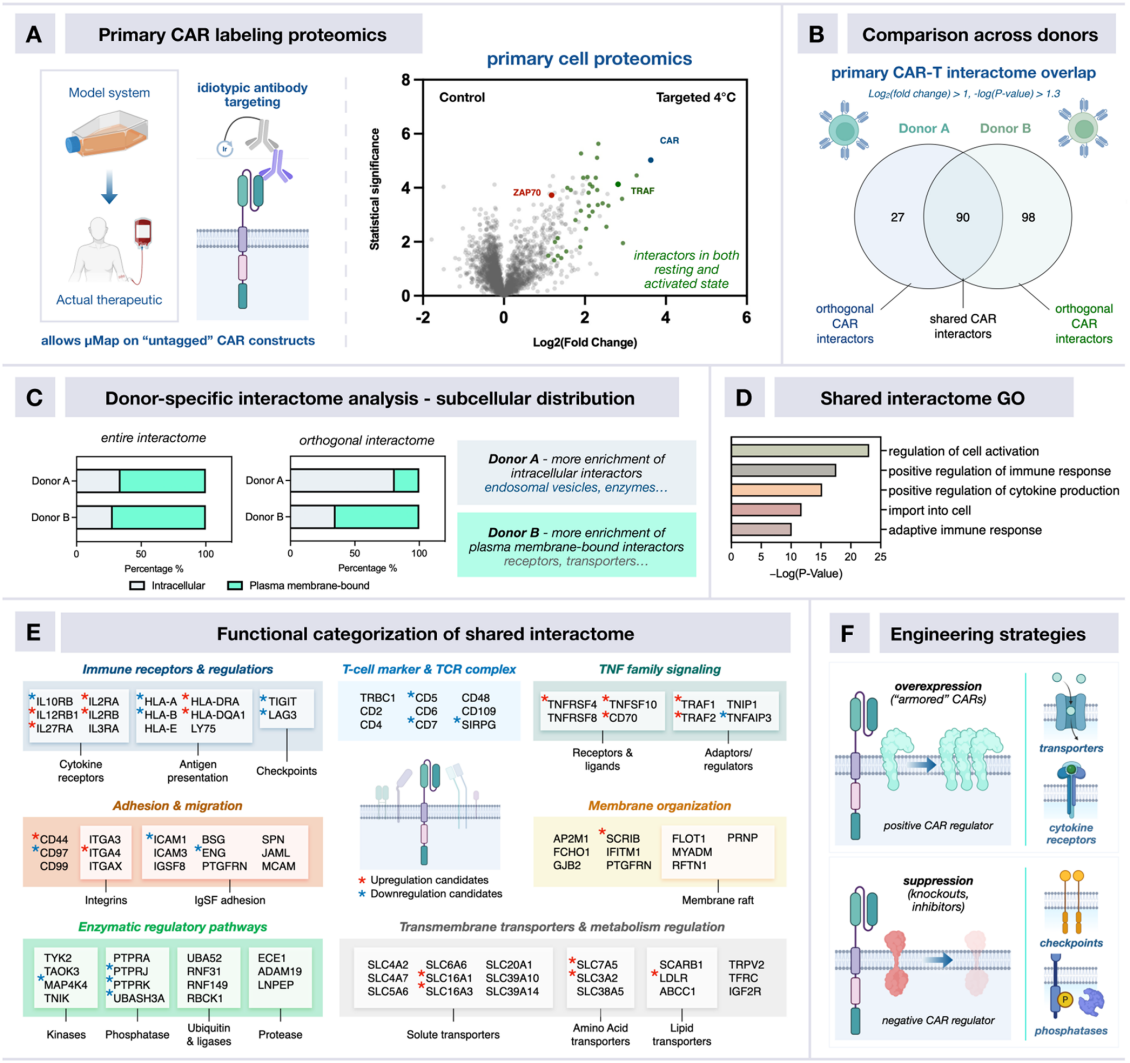
(a) Schematic of μMap-CAR labeling of primary CAR-T using an idiotypic antibody to target the untagged CAR. The primary CAR interactome (Donor A) illustrates canonical CAR signaling interactors (Log_2_(Fold Change)>1, −Log­(P-value)>1.3, two-tailed Student’s *t*-test, triplicate in each condition). Green color marks interactors present in both resting and activated states. (b) A Venn diagram of enriched hits from two donors demonstrates orthogonal and shared interactors. (c) Donor-specific interactome analysis reveals differences in subcellular distribution and functional categories. (d) Gene ontology analysis of overlapping hits reveals the top-enriched pathways to be closely correlated with CAR-T function. (e) High-fidelity shared CAR interactome (*n* = 90) across the two donors was grouped into various functional categories important to T-cell function. Red stars denote representative candidates whose enhancement is predicted/examined to improve TCR/CAR performance, whereas blue stars denote inhibitory candidates suitable for suppression. (f) Conceptual schematic outlining proposed future CAR-T engineering approaches guided by interactome-derived candidate targets.

Following a similar labeling workflow, we profiled primary CAR-T cells from two healthy donors, A and B, with triplicate samples examined to improve data fidelity (Figure 5A-B, Figures S8, S9). As expected, differences were observed between the individual donor interactomes, revealing donor-dependent differences in interactome size and subcellular localization (Figure 5C).^48,49^ Remarkably, this comparison also revealed 90 proteins shared by both CAR interactomes, significantly exceeding random expectation (hypergeometric test, *p* < 10^–90^). GO analysis of this “core” CAR interactome highlighted major pathways related to cellular activation and immune responses, plausibly representing core components of the microenvironment (Figure 5D).

We next mapped this “core” CAR interactome—composed of both known and previously unknown TCR/CAR interactor proteins—to a variety of cellular functions related to T-cell signaling and regulation (Figure 5E). As expected, we identified canonical TCR complex, T-cell marker, and TNF signaling proteins, which are key to CAR activation and inform CAR design principles from the TCR complex.^35^ We also identified surface receptors involved in other immune regulation pathways, such as the human leukocyte antigen (HLA) complex, which has been engineered in allogeneic CAR-T therapy to extend the durability of CAR-T therapy in the patient.^50^ Additionally, we identified cytokine receptors and canonical immune checkpoints, interactors that are rarely captured in our Jurkat model system, underscoring specific CAR features derived from exposure to the *in vivo* plasma environment.^51,52^ Some of these proteins, including IL2RA/B and TIGIT, have been explored in CAR engineering and combinatorial therapeutic strategies to improve CAR-T cell potency.^53,54^

Beyond these surface immune receptors and coregulators, we discovered additional proteins that may facilitate immune regulation of CAR-T. These include a variety of adhesion and membrane organization proteins, which may assist in T-cell recognition and activation or contribute to CAR internalization and recycling.^55,56^ Intriguingly, a set of membrane transporters for amino acids, lipids, and other solutes was also identified, offering insights into the metabolic regulation of CAR-T cell activation—an active area of CAR engineering.^57^ T-cell signaling relies on finely tuned post-translational modifications, such as phosphorylation and ubiquitination, and we identified a range of related downstream enzymes, including kinases, phosphatases, and ubiquitin ligases.^58–60^

Importantly, many of the enriched targets align with ongoing preclinical and clinical efforts to improve CAR performance through functional enhancement (e.g., overexpression or armored-CAR strategies) or inhibition (e.g., inhibitors or knockout approaches) (Figure 5E-F).^61^ For instance, metabolic and membrane transport proteins frequently correspond to overexpression targets, supporting CAR-T expansion and activation. By contrast, negative signaling regulators, including phosphatases that restrain CAR signaling, represent inhibitory nodes whose attenuation may overcome insufficient activation.^62,63^ There is also a subset of proteins representing gene perturbation screening hits in CAR-T contexts, including LAG3, TNIK, SLC16A1, TNFAIP3, UBASH3A, and TRAF1 (Figure S10).^15–17^ We believe that this comprehensive profile of the CAR microenvironment offers a valuable resource for advancing our understanding of basic CAR biology and informing next-generation CAR-T engineering.

### 2.4 Conserved CAR-Proximal Proteins Function as Regulators of CAR Signaling

Independent profiling of the CAR interactome in Jurkat cells and primary human CAR-T cells revealed substantial overlap (n = 55 hits) between the two systems, despite context-specific differences (Figure S11). This convergence indicates that μMap-CAR robustly captures conserved features of the CAR microenvironment across both model and physiologically relevant settings. To validate these findings, we confirmed the presence of several previously underexplored hits in the CAR microenvironment using super-resolution STED microscopy (Figure 6A). Validated hits include the T-cell surface marker CD7; the amino acid transporter SLC7A5; proteins involved in adhesion and cell membrane organization, such as ITGA4, CD97/ADGRE5, and RFTN1; and phosphorylation regulators such as the phosphatase PTPRA and the kinase TYK2. Optimal signal overlap was observed for certain membrane-bound proteins, including SLC7A5 and PTPRA, while partial colocalization was observed for cytoplasmic proteins such as TYK2. To further explore the functional connection between selected hits and CAR signaling, we performed siRNA-mediated knockdown of PTPRA and TYK2 (Figure 6B). Nucleofection was employed to enable efficient siRNA transfection with minimal toxicity (Figure S12). Partial loss of protein levels resulted in increased phospho-ZAP70 and/or phospho-ERK levels upon antigen exposure in a time-dependent manner: TYK2 knockdown increased pZAP70 and pERK, with maximal effects observed at 30 min (Figure 6B), whereas PTPRA knockdown showed a more pronounced increase in pZAP70 at 1 h, consistent with a time-dependent accumulation of phosphorylation (Figure S12). These observations suggest that these proteins may function as modulators that restrain CAR signaling and highlight the complex regulatory landscape of phosphorylation signaling networks, wherein multiple kinases, phosphatases, and cytokine-linked pathways collectively shape CAR activation dynamics.^59,64^ Notably, regulators within these signaling axes—including kinase and phosphatase nodes analogous to PTPRA and TYK2—are increasingly being explored as targets in CAR-T engineering to tune activation strength and persistence.^65^ Collectively, these colocalization patterns and gene perturbation phenotypes not only validate that μMap-CAR captures the CAR microenvironment, but also demonstrate its ability to identify functionally relevant and potentially engineerable nodes within CAR signaling networks.

**Figure 6:**
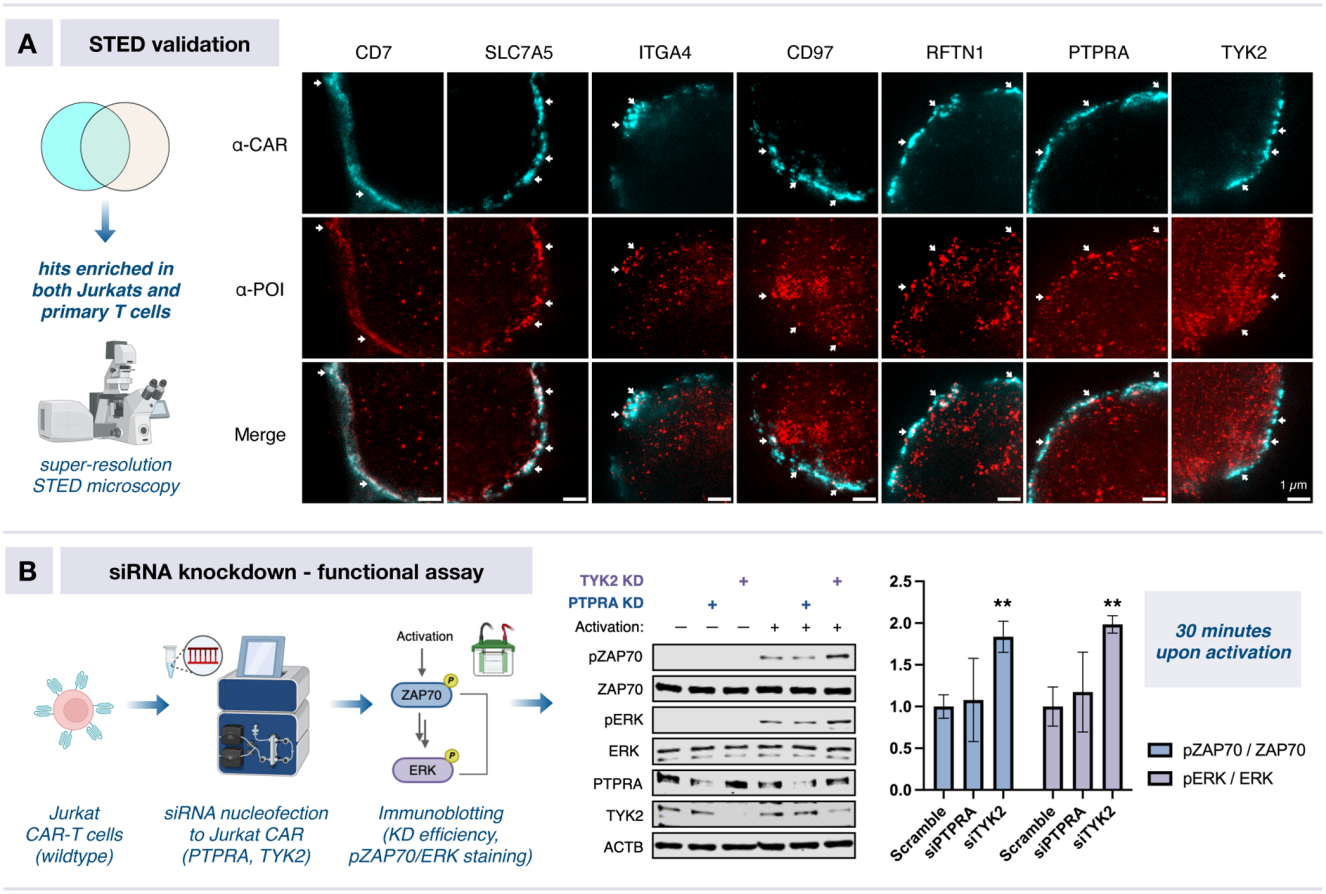
(a) Selected proteins of interest (POI) from high-fidelity CAR interactors robustly captured across Jurkat and primary CAR-T cells are validated as colocalizing with CAR constructs via super-resolution STED microscopy in Jurkat CAR-T cells. Cyan, signal of anti-CAR idiotypic antibody; red, signal of anti-POI listed in each column title; white, signal overlap, further indicated with white arrows. Results were validated via independent repeats. (b) Functional perturbation of CAR-associated regulators. Jurkat CAR-T cells were nucleofected with siRNAs targeting PTPRA or TYK2, followed by CAR activation and immunoblotting. Quantification of pZAP70/ZAP70 and pERK/ERK intensity 30 min upon activation is shown (biological triplicates; **P < 0.01, one-way ANOVA, mean ± s.d.).

## 3 Discussion

In conclusion, we present μMap-CAR, a high-resolution photocatalytic labeling platform capable of mapping functional CAR interactomes across Jurkat model and primary T-cell systems. Importantly, this platform incorporates substantial methodological advances, including optimization of the labeling radius and an activation-locking workflow to preserve transient CAR-associated complexes for reproducible profiling. In Jurkat model systems, μMap-CAR selectively resolves altered interactome signatures arising from CAR endodomain perturbations, enabling, for the first time, precise resolution of mutation-driven interactome differences. Extending this approach to primary human CAR-T cells, we obtained high-quality interactor data sets across two independent healthy donors, demonstrating the first comprehensive intrinsic CAR interactome as a valuable resource for future studies.

The data sets obtained using μMap-CAR provide insight into the molecular determinants that influence CAR signaling and effectiveness, highlighting novel engineerable targets for CAR-T optimization. Across Jurkat models, mutant constructs, and donor-derived CAR-T cells, μMap-CAR consistently identified a shared core interactome, suggesting the presence of conserved molecular components that form the regulatory architecture surrounding CAR signaling. Within these data sets, established CAR signaling relationships were readily recapitulated: disruption of CD3ζ ITAM motifs selectively diminished enrichment of the ZAP70–GRB2/CRKL signaling axis, while mutation of the 4–1BB costimulatory domain abolished recruitment of the TRAF2/5–BIRC2/3 module, consistent with their canonical roles in proximal activation signaling and CAR-T persistence. Beyond these expected relationships, the interactome revealed additional regulators within the CAR microenvironment, including proteins implicated in mechanosensitive signaling and cytoskeletal remodeling (e.g., PIEZO1, ICAM3, and FNBP1), as well as signaling modulators such as PTPRA and TYK2, whose perturbation enhances phospho-ZAP70 levels upon activation. Notably, several proteins identified by μMap-CAR overlap with targets currently being explored in CAR-T engineering, including metabolic transporters and immune regulators. Yet most, including PTPRA and TYK2, were not previously known to directly interact with the CAR, underscoring the potential for μMap-CAR to uncover novel CAR engineering targets.

We note that surface-proximal interactome profiling provides a theoretically unbiased view of CAR-associated molecular neighborhoods, which may not always be readily attributable to specific donor characteristics or cellular phenotypes defined by genomic or transcriptional features. Donor-to-donor variability in T-cell state, signaling capacity, or differentiation history may influence certain aspects of CAR-associated interactions; however, the striking overlap observed between primary CAR-T interactomes across donors—and with the Jurkat model—underscores the presence of a conserved core CAR microenvironment. This convergence supports the existence of shared, intrinsic CAR biology that is robust to cellular context, while highlighting the potential for context-dependent modulation at the periphery of this core network. μMap-CAR thus delivers the first high-resolution CAR intrinsic interactome map, providing a resource that we anticipate will inform the next generation of CAR engineering efforts.

In future studies, we aim to leverage the modular nature of μMap-CAR to study CARs with alternate targeting motifs (ScFv, such as against BCMA) or costimulatory domains; examine context-dependent variation across a broader donor population, including age, gender, CD4:8 ratio, T cell state, and CAR expression; interrogate CAR-T tumor interactions in primary patient-derived T-cells; and integrate interactome profiling of distinct CAR-T subtypes to inform next-generation therapeutic design.

## Supplementary Material















## Data Availability

Proteomics raw data have been uploaded to the MASSive database: accession number MSV000101544, the dataset is now public: http://massive-ftp.ucsd.edu/v12/MSV000101544/.

## Notes

Experimental procedures and supplementary figures (PDF)
Tabulated proteomics data of Jurkat CAR interactome (XLSX)
Tabulated CAR wild-type interactome analysis and gene annotations (XLSX)
Tabulated CAR mutant interactome analysis and gene annotations (XLSX)
Tabulated global proteomics data of CAR mutant (XLSX)
Tabulated proteomics data of primary CAR interactome (XLSX)
Tabulated primary CAR cross-donor analysis and gene annotations (XLSX)
