# Supplementary material for: High-Precision Intrinsic Interactome Elucidation of Chimeric Antigen Receptors via Photocatalytic Micromapping (μMap-CAR)
Source: J Am Chem Soc. 2026 Jul 1;148(27):29361–71. doi: 10.1021/jacs.6c08969 (PMC13383733; doi:10.1021/jacs.6c08969)
Supplement: Supplementary file 1 [file ja6c08969_si_001.pdf]

High-precision Intrinsic Interactome Elucidation of Chimeric Antigen Receptors via Photocatalytic Micromapping (µMap-CAR)

Supporting Information

Sean W. Huth,[1,2,‡] Chenmengxiao (Roderick) Pan,[1,2,‡] Philip Raftopoulos,[1,2] Ciaran P. Seath,[1,2] Gabrielle H. Lovett,[1,2] Beryl Li,[1,2] Sushma Yechan Gunja,[3] Vaishali Agarwal,[3] Yuka Amako,[4] Harris Bell-Temin,[4] Helen Pham,[4] Helen Evans,[4] Jennifer X. Qiao,[4] Haibo Liu,[4] Brook Barajas,[3] and David W.C. MacMillan[1,2,*]

[1]Merck Center for Catalysis at Princeton University, Princeton, NJ 08544, USA

[2]Department of Chemistry, Princeton University, Princeton, NJ 08544, USA

[3]Bristol Myers Squibb, Seattle, WA 98109, USA

[4]Discovery & Development Sciences, Bristol Myers Squibb, Cambridge, MA 02141, USA

[*]Corresponding author. Email: dmacmill@princeton.edu.

[‡]S.W.H. and C.(R.)P. contributed equally to this work

TABLE OF CONTENTS

Supporting Figures S3

Chemical Structures S13

General Materials S14

General Procedures S16

Proteomics Principal Component Analysis (PCA) S25

STED Microscopy Uncropped Images S26

CAR sequence S28

Donor information S29

References S30

Supporting Figures

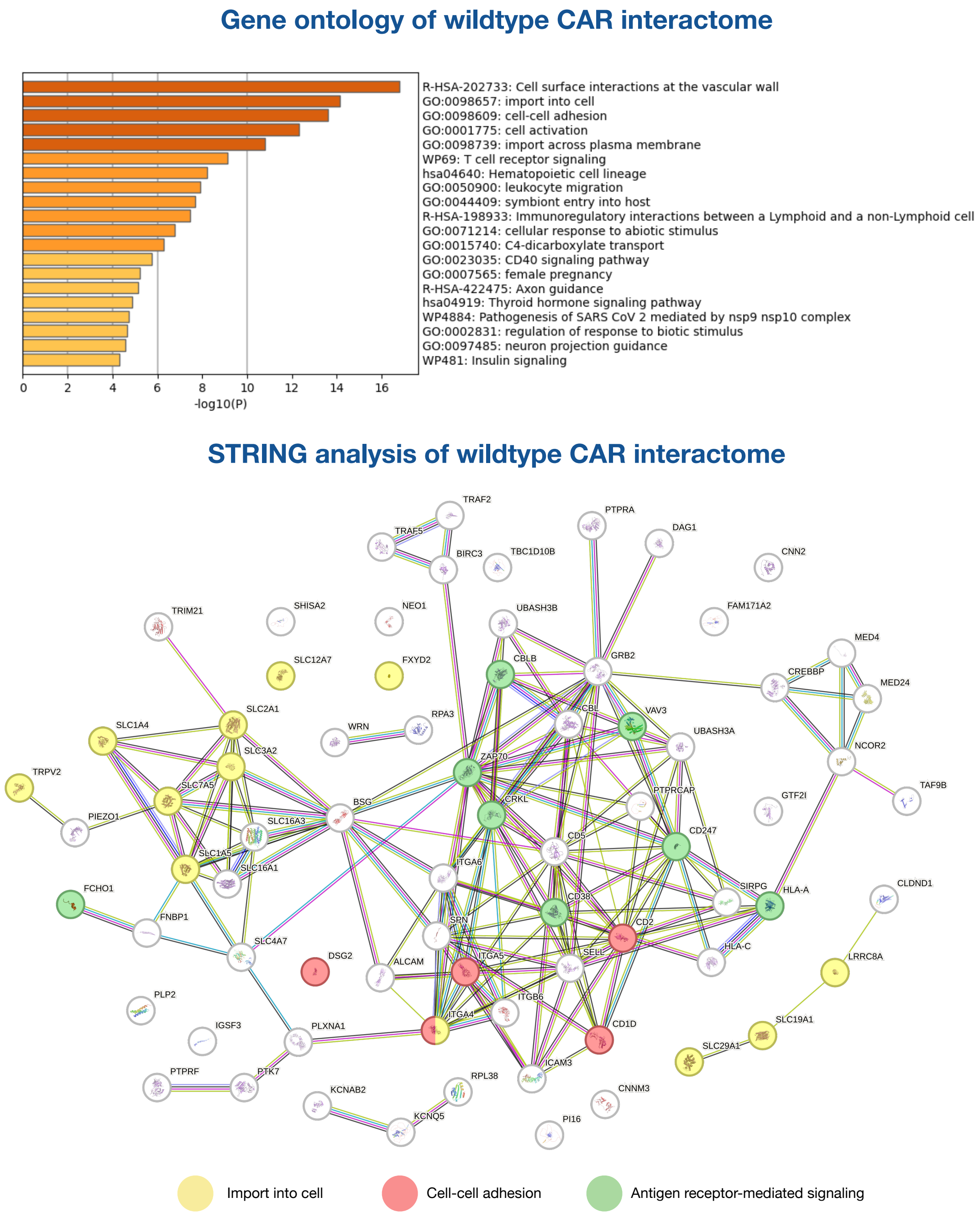

Figure S1: Gene ontology and STRING analysis of top interactors (Log_2(fold change) > 1, −log(P-value) > 1.3) enriched in Jurkat CAR labeling dataset.

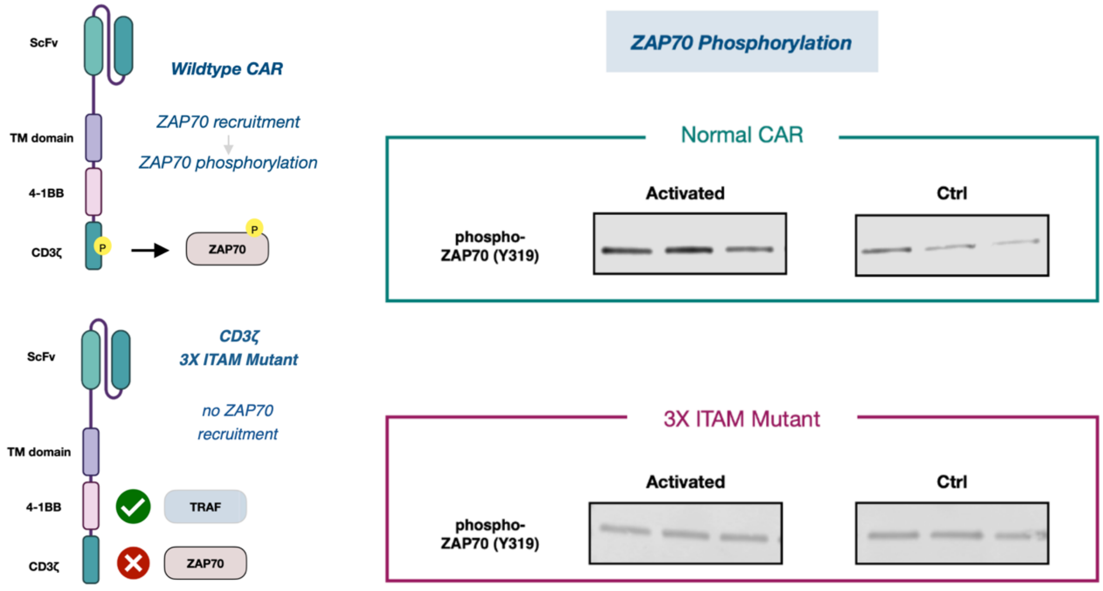

Figure S2: Validation of 3× ITAM mutant signaling being repressed via phospho-ZAP70 staining.

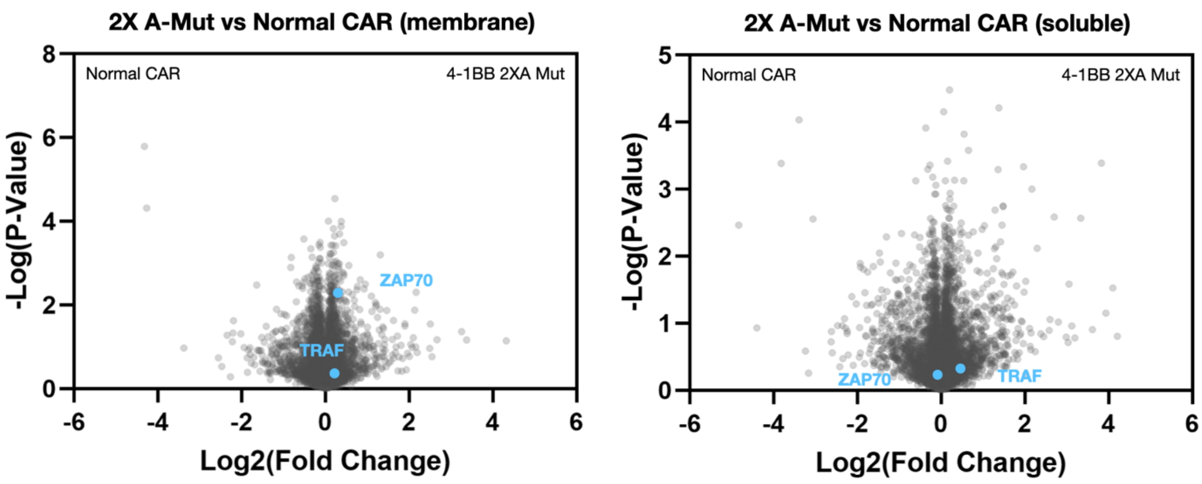

Figure S3: Global proteomics comparing 4-1BB 2× A Mutant CAR with normal CAR-expressing Jurkat cells in the membrane fraction and intracellular soluble fraction. TRAF5 and ZAP70 are highlighted on each plot, demonstrating minimal perturbation to the protein level.

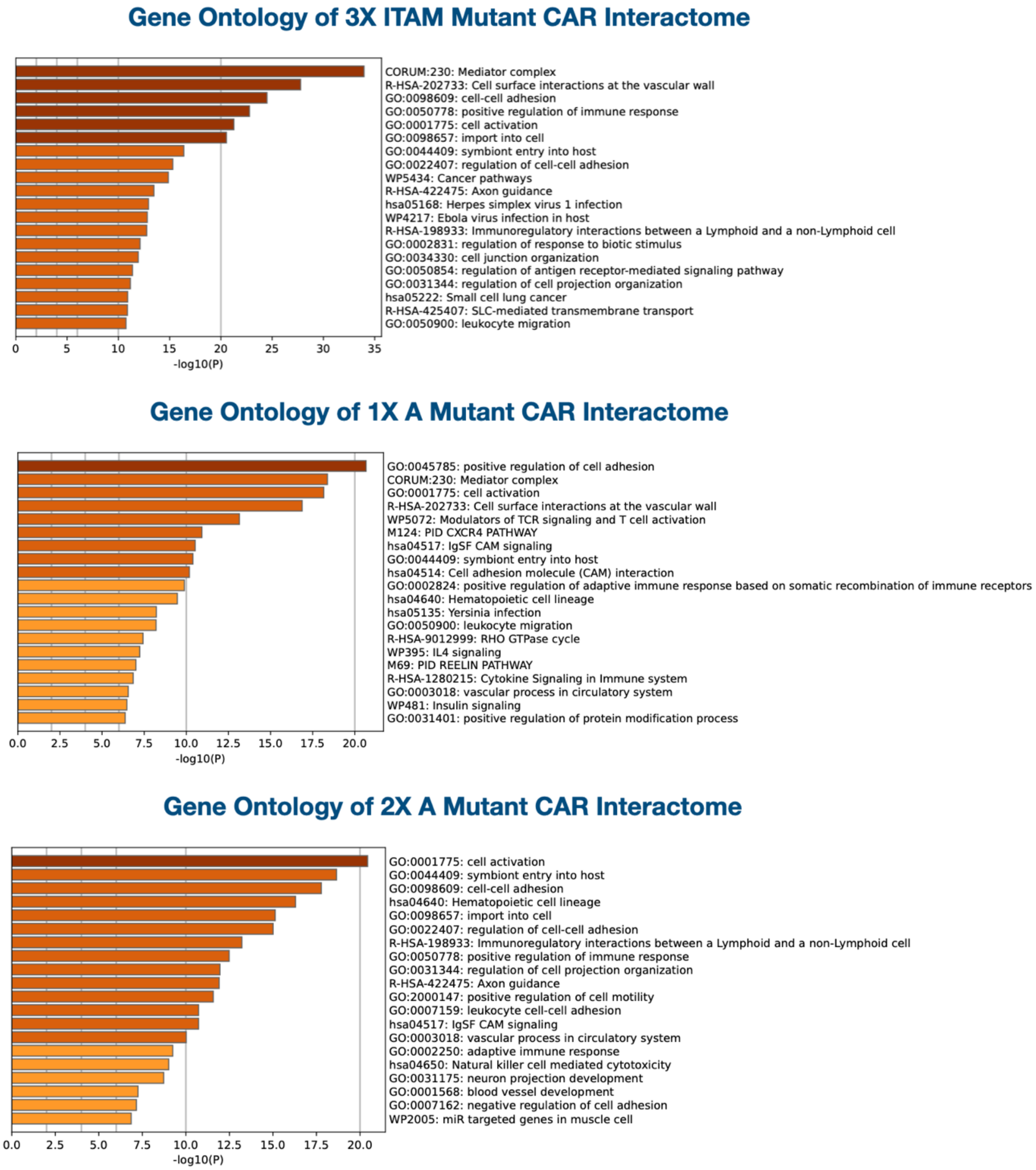

Figure S4: Gene ontology analysis of top enriched interactors (Log_2(fold change) > 1, −log(P-value) > 1.3) in the CD3ζ 3× ITAM mutant, 4-1BB 1× A mutant, and 4-1BB 2× A mutant datasets.

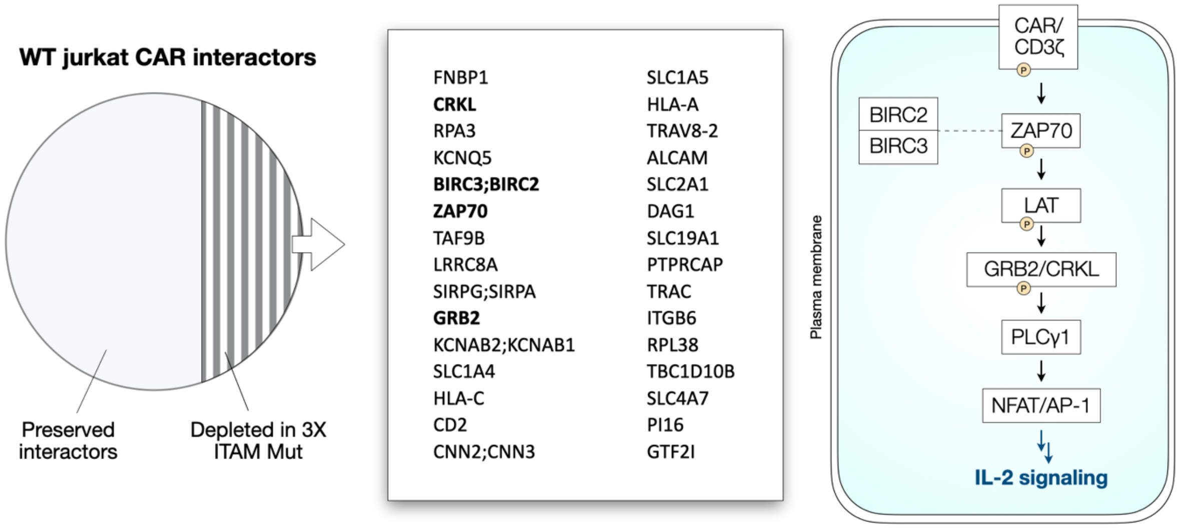

Figure S5: Interactors present in the WT CAR microenvironment but reduced upon 3× ITAM mutation, highlighting disruption of the CD3ζ–ZAP70–GRB2/CRKL signaling axis and downstream IL-2 activation pathways.

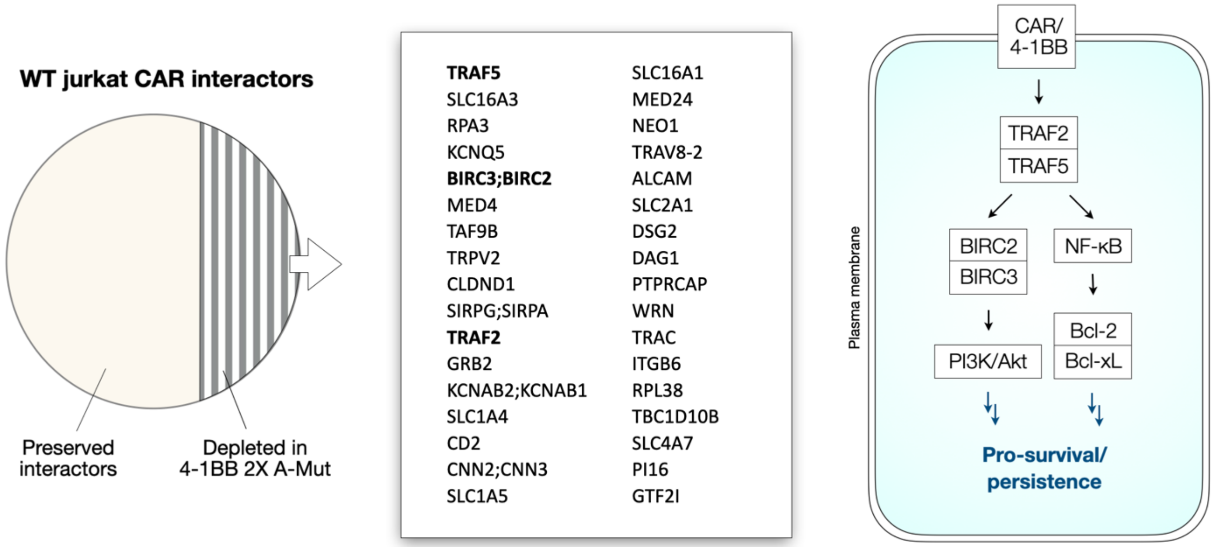

Figure S6: Interactors present in the WT CAR microenvironment but reduced upon 4-1BB 2× A mutation, highlighting disruption of the canonical 4-1BB–TRAF2/5–BIRC2/3 costimulatory axis and downstream Akt-associated pro-survival and metabolic support pathways.

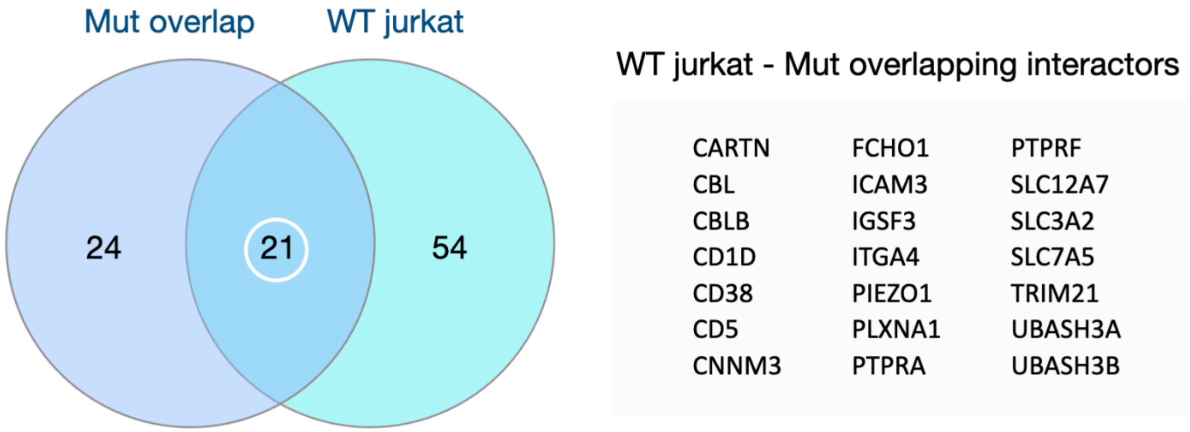

Figure S7: Interactors overlap diagram comparing interactors enriched in all three mutants and wildtype Jurkat CAR labeling.

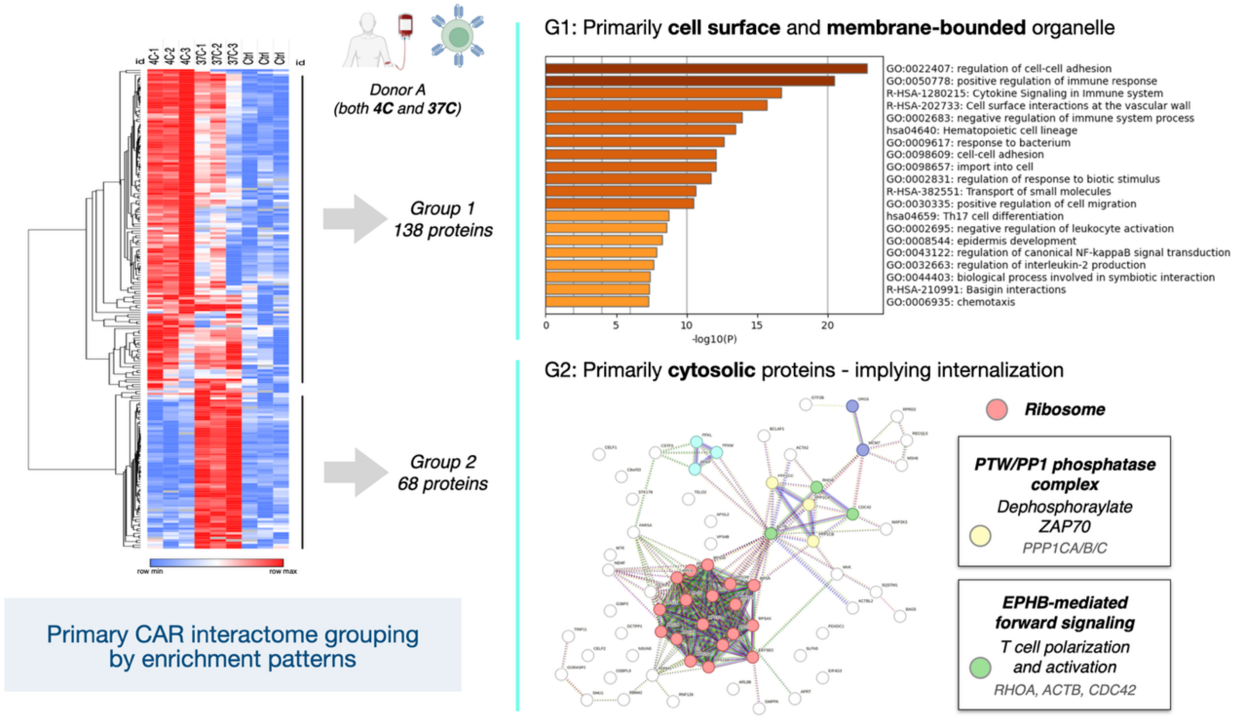

Figure S8: Heatmap grouping of primary CAR-T cell hits from donor A labeling data (4 ºC "resting" state and 37 ºC "activated" state CAR interactome (Log_2(fold change) > 1, −log(P-value) > 1.3) reveals overlapping and preferential enrichment of two groups of proteins. Red, high protein enrichment intensity; blue, low intensity. Group 1 primary cell protein gene ontology reveals they are primarily surface proteins and correspond to expected T-cell function; Group 2 protein gene ontology reveals T-cell cytosolic signaling pathways.

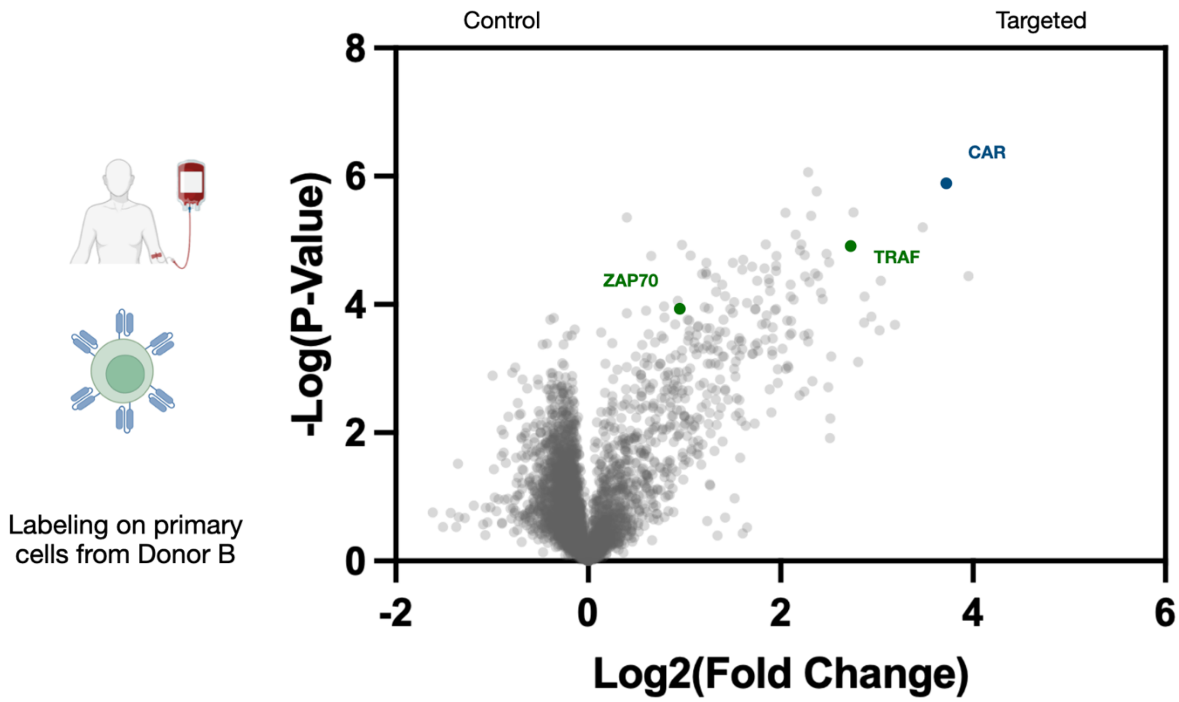

Figure S9. Volcano plot proteomics data of primary CAR-T labeling, Donor B. CAR, TRAF, and ZAP70 were highlighted on the plot.

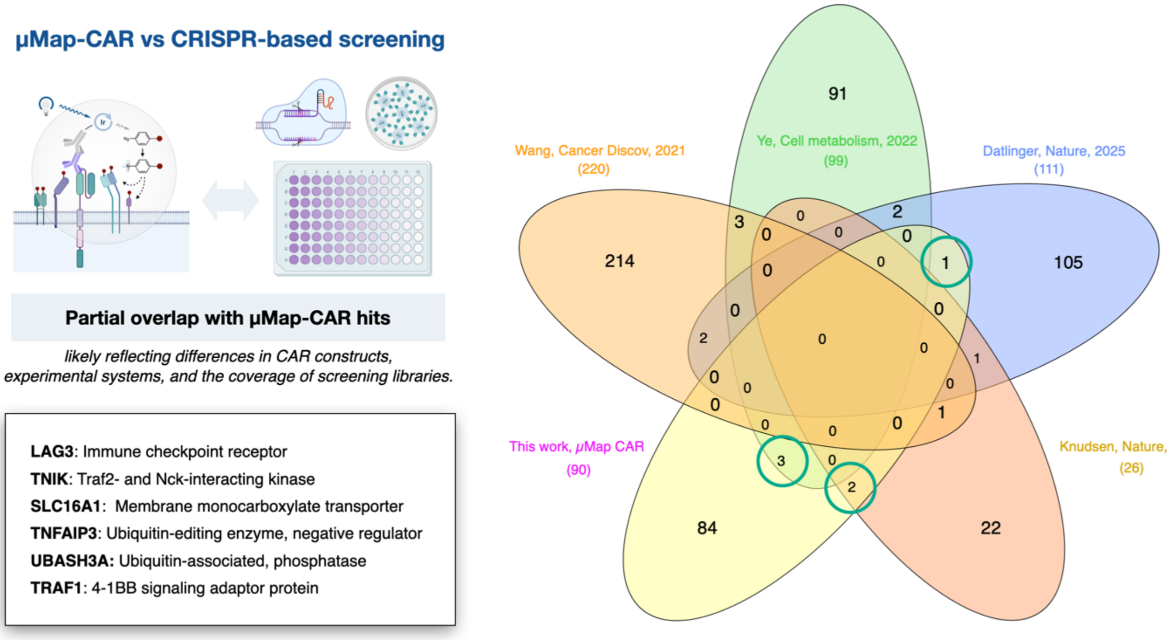

Figure S10. A subset of proteins identified by μMap-CAR overlaps with regulators previously discovered through CAR-T gene perturbation screens, including LAG3, TNIK, SLC16A1, TNFAIP3, UBASH3A, and TRAF1, suggesting partial convergence between interactome profiling and functional genetic approaches.

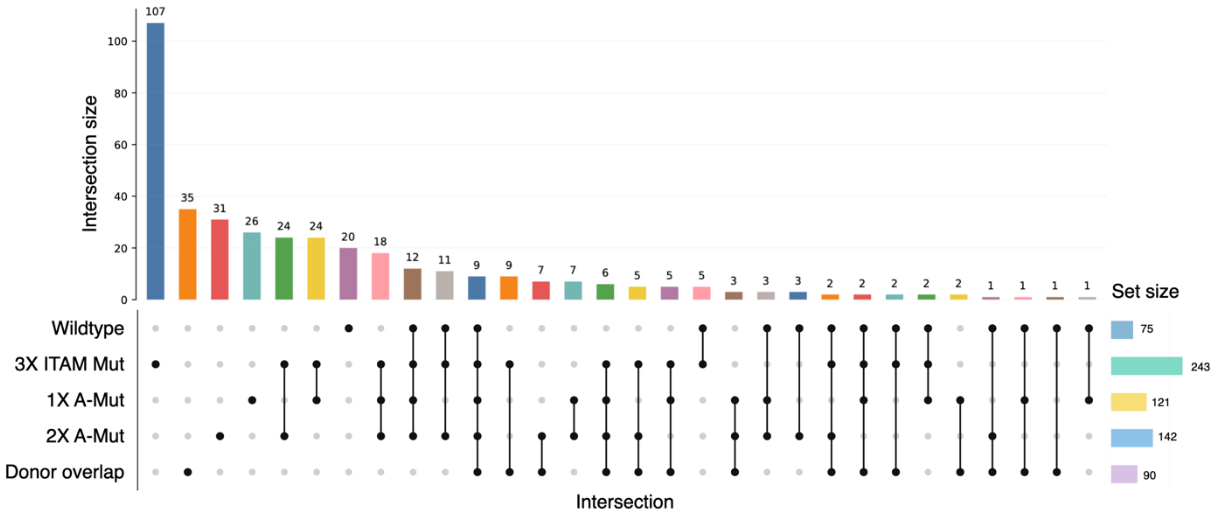

Jurkat–primary shared CAR interactome

ABCC1	CD6	IGF2R	PRNP	SIRPG	SLC5A6	TNIP1
ADGRE5	CD7	IGSF8	PTPRA	SLC16A1	SLC6A6	TRAF1
AP2M1	CD70	ITGA3	PTPRJ	SLC16A3	SLC7A5	TRAF2
BSG	ECE1	ITGA4	RBCK1	SLC38A5	SPN	TRBC1
CARTN	FCHO1	LDLR	RFTN1	SLC39A10	TAOK3	TRPV2
CD2	HLA-A	LNPEP	RNF149	SLC3A2	TFRC	TYK2
CD4	ICAM1	MAP4K4	RNF31	SLC4A2	TNFAIP3	UBASH3A
CD5	ICAM3	MYADM	SCARB1	SLC4A7	TNFRSF4	

Figure S11. Overlapping diagram of interactors enriched across Jurkat and primary CAR-T cells. The shared interactome between Jurkat and primary CAR-T cells is listed (n = 55 hits).

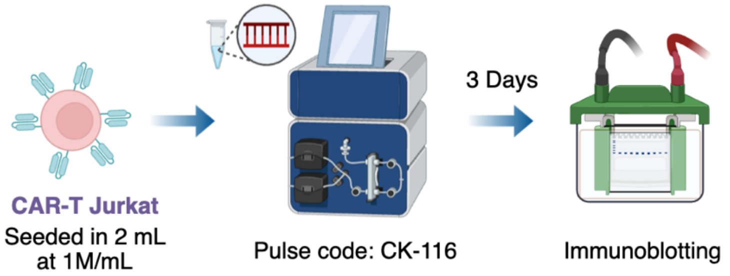

Viability test after transfection

	Viability percent	Cell count (per mL) after 3 days
**Scr (control)**	96%	1.84 M
**PTPRA**	96%	1.94 M
**TYK2**	97%	1.90 M

1 hour upon activation

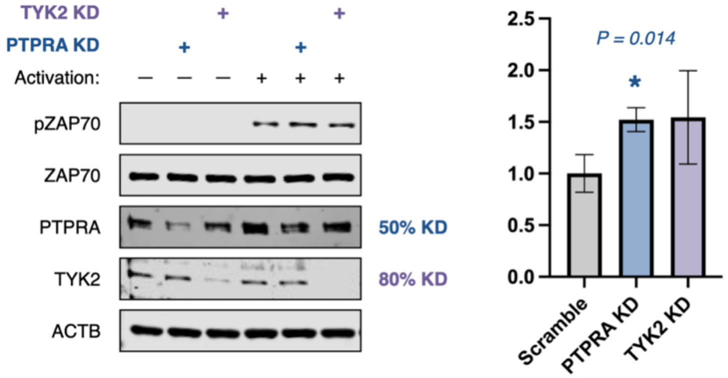

Figure S12. CAR-T Jurkat cells transfected with siRNAs targeting PTPRA or TYK2 showed comparable viability and cell counts after 3 days to the scrambled siRNA control. Immunoblotting (biological triplicates) following 1 hour of CAR activation showed increased pZAP70 levels upon knockdown of either gene.

Chemical Structures

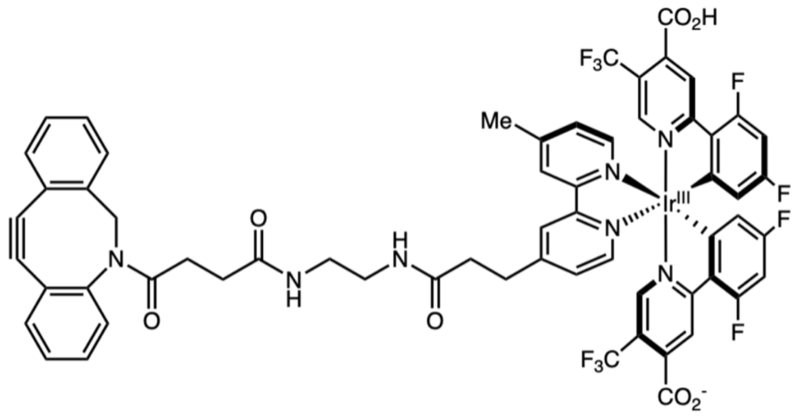

Iridium(G3)-DBCO **(S1)**

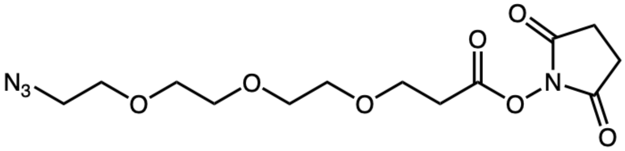

Azido-PEG$_3$-NHS **(S2)**

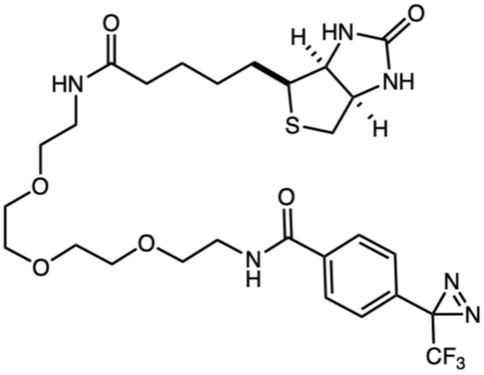

Biotin-PEG$_3$-Diazirine **(S3)**

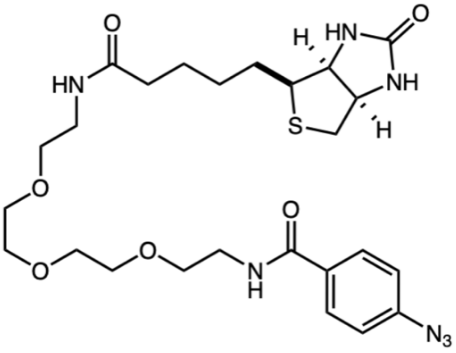

Biotin-PEG$_3$-Phenyl Azide **(S4)**

General Materials

All buffers and synthetic starting materials were used as received from commercial sources. Bovine serum albumin (BSA) (A7906), Eppendorf Protein LoBind tubes (Z666505), iBright Prestained Protein ladder (LC5615) were purchased from Thermo Scientific (Rockford, IL). TBST (IBB–581X) was purchased from Boston BioProducts (Ashland, MA). 12% Criterion TGX precast gels (5671044) and 4x Laemmli sample buffer (161–0747) were purchased from Bio–Rad (Hercules, CA). 20% SDS solution (351–066–721) was purchased from Quality Biological (Gaithersburg, MD). Ir-G3-DBCO (**S1**), Biotin-PEG3-Diazirine (**S3**), and Biotin-PEG3-Phenyl-Azide (**S4**) were prepared as previously reported.[1,2] Azido-PEG3-NHS (**S2**) was purchased from BroadPharm.

Cell Lines

Jurkat (TIB-152) cells were purchased from American Type Culture Collection (ATCC), and cultured in GlutaMAX RPMI (Gibco, #61870036), with 10% FBS (Gibco, #10437-028), supplemented with 1% Penicillin/Streptomycin (Gibco, #15070063) at 37°C, 5% CO_2. Jurkat/primary CAR-T cells were generated and provided by BMS.

Antibodies

Anti-HA: abcam, ab9110, rabbit

Idiotypic antibody: mouse, provided by BMS

Anti-PIEZO1: Invitrogen, PA5-106296, rabbit

Anti-ZAP70: Cell Signaling Technology, 2705S, rabbit

Anti-pZAP70: Cell Signaling Technology, 2701S, rabbit

Anti-ERK: Santa Cruz Biotechnology, sc-514302, mouse

Anti-pERK: Cell Signaling Technology, 4370S, rabbit

Anti-CD7: Cell Signaling Technology, 32814T, rabbit

Anti-SLC7A5: Proteintech, 28670-1-AP, rabbit

Anti-PTPRA: Proteintech, 13079-1-AP, rabbit

Anti-TYK2: Proteintech, 83161-4-RR, rabbit

Anti-ITGA4: Proteintech, 19676-1-AP, rabbit

Anti-CD97: Invitrogen, MA5-53469, rabbit

Anti-RFTN1: Proteintech, 24289-1-AP, rabbit

Anti-ACTB: Cell Signaling Technology, 8457S, rabbit

Goat-anti-rabbit-STAR Red: Abberior, STRED-1002-500UG

Goat-anti-mouse-STAR Orange: Abberior, STORANGE-1001-500UG

General Procedures

CAR-T Cell Generation

Jurkat CAR-T cells were generated by transducing with lentiviral particles via spinoculation in a 96-well format. Cells were cultured in complete GlutaMAX RPMI 1640 medium supplemented with 10% fetal bovine serum (FBS), 1% sodium pyruvate, and 1% MEM non-essential amino acids. Lentiviral titration was performed by preparing 2-fold serial dilutions in a U-bottom 96-well plate using pre-warmed RPMI. Protamine sulfate was added to each well to a final concentration of 10 μg/mL. Jurkat cells were harvested, pelleted, and resuspended at 5×10^6 cells/mL in complete medium. 100 μL of the cell suspension (5×10^5 cells) was added per well to the prepared viral dilutions. Plates were centrifuged at 1200 × g for 30 min at 32 °C. Following spinoculation, the transduction mixture was gently removed, and cells were resuspended in 100 μL of fresh medium, then transferred to 24-well plates pre-filled with 500 μL of warm medium. Transduced cells were cultured at 37 °C, and the complete RPMI medium was refreshed every other day until Day 7 for cryopreservation. Transduction was analyzed by flow cytometry on Day 3.

To generate primary CAR-T cells from human donors, primary $CD4^+$ and $CD8^+$ T cells were thawed, pooled at a 1:1 ratio, and stimulated with Expamer (final concentration 12 μg/mL) in 2D25G medium (OpTmizer basal medium supplemented with ICSR, GlutaMAX, and cytokines IL-2, IL-7, IL-15) at a final concentration of 3×10^6 cells/mL. 24 hours after stimulation, cells were collected, counted, and resuspended at 5×10^6 cells/mL. Transduction was performed by spinoculation with pre-titrated lentivirus in non-TC plates. Virus was added directly to medium and cells (typically 5×10^5 cells per well in 96-well plates, total volume 200 μL), followed by centrifugation at 1000 × g for 30 min at 30 °C. After spin, cells were transferred to 24-well plates containing medium supplemented with cytokines and Expamer and cultured overnight. Expamer was inactivated on Day 3 by replacing the medium with D-biotin–containing medium (1 mM final). Cells were maintained for up to 9 days with cytokine-supplemented medium exchanges every other day. Phenotypic and functional assessments were performed by flow cytometry on Days 4 and 7, and cells were harvested on Day 9 for cryopreservation.

CAR-T Cell Activation Reporter Assay

Two Jurkat reporter cell lines were used to enable evaluation of CAR signaling in the presence of anti-CAR or anti-HA antibody. The first cell line contained a standard NFkB reporter comprising concatenated NFkB transcription factor binding sites upstream of a minimal promoter and tdTomato fluorescent protein, delivered via retrotransposon into Jurkats, which were then clonally expanded. The second contained a tdTomato reporter knocked into the Nur77/NR4A1 gene locus via CRISPR/Cas9 editing, under control of the CD3-responsive native Nur77/NR4A1 promoter. These Jurkat reporter lines were lentivirally transduced with C-terminally HA-tagged anti-CD19 CAR as described above.

For the activation assays, 2×10^4 transduced Jurkat CAR T cells were plated in complete RPMI in 24-well cell-culture plates at a 1:1 ratio with a CD19-overexpressing K562 cell line (CD19+), or a K562 line without CD19 (CD19-). These co-cultures were treated with 5 µg/mL Abcam anti-HA antibody (ab9110) or no antibody and incubated at either 4 °C or 37 °C overnight. Flow cytometry was run to evaluate CAR expression using an anti-CAR antibody (α-ID-AF467 (1:1000)), or CAR signaling through a reporter (tdTomato read through the PE channel). In brief, live cells were aliquoted into a 96-well plate, pelleted at 1,000 g for 1 minute, and stained with Near-IR live/dead distinguishing cell dye (Thermo Fisher Scientific, catalog# L10119). Cells were then washed and stained according to standard surface staining procedure in Cell Staining Buffer (BioLegend, catalog# 420201). Numbers of tdTomato+ cells were reported as the percent of positive cells out of the total number of cells enumerated by flow from each sample.

Procedure for CAR-T Proteomics Labeling

For targeted arms, 10 million CAR-T cells per replicate were resuspended in 1 mL cold DPBS containing 5 µg HA (for Jurkat) or idiotypic antibody (for primary cells) and incubated at either 4 °C for 1 hour, which is sufficient for antibody-receptor engagement at resting states, or 37 °C for 1 hour to allow receptor engagement and formation of CAR-associated signaling complexes. After incubation, cells were rapidly cooled to preserve the surface CAR interactome, and washed 3× with ice-cold DPBS before being resuspended in 1 mL DPBS with 2.5 µg secondary iridium conjugate antibody (HA – rabbit, idiotypic – mouse) following published protocols.[1,3] After 1

further hour incubation at 4 °C, cells were washed 3× with cold DPBS and resuspended in 1 mL DPBS containing 250 µM Diazirine or Phenyl-Azide probe. Cells were then irradiated for 3 minutes at 100% LED intensity in an M2 Integrated Photoreactor at 4 °C. Upon irradiation, cells were washed 2× with cold DPBS before being pelleted and frozen for further downstream processing. For control arms, all steps are the same except that cells are incubated without primary antibodies at 4 °C for 1 hour before secondary antibody incubation.

Cells were resuspended in 500 µL of memPER membrane permeabilization buffer. After 20 minutes of incubation at 4 °C, the resulting mixture was centrifuged at 10000 g for 10 minutes to isolate the membrane fraction as a pellet. This pellet was resuspended in 500 µL RIPA containing 1% SDS and cOmplete protease inhibitor. Lysates were then sonicated in the Bioruptor for 10 minutes (high power, water bath 4 °C, 15 seconds on/15 seconds off). Cells were boiled for 10 minutes at 95 °C. After heating, another 600 µL of RIPA with cOmplete was added. Resulting lysates were pelleted at 15000×g for 10 minutes, and the resulting supernatant was transferred to a new tube to clarify lysates. Protein concentrations were measured via BCA assay.

After BCA, 1 mg of total protein per replicate was loaded onto 100 µL Thermo streptavidin beads and incubated at 4 °C overnight for pulldown. The next day, proteins were reduced via 10 mM DTT at room temperature for 20 minutes, before being alkylated with 15 mM IAA for 30 minutes in the dark at room temperature. Finally, IAA was quenched by adding 20 mM DTT for 15 minutes at room temperature. After alkylation and reduction, beads were washed 3× with DPBS containing 1% SDS, 3× with DPBS containing 1 M NaCl, and 3× with DPBS containing 10% ethanol. Following these washes, beads were washed 3× with 50 mM Ammonium Bicarbonate Buffer in Optima Water.

For Western blot analysis, beads were resuspended in DPBS in new LoBind tubes, pelleted, and incubated with 30 µL freshly made elution buffer (30 mM biotin, 6 M urea, 2 M thiourea, 2% SDS in DPBS, 20% 4x Laemmli with BME, pH = 11.5) and heated to 95 ºC for 15 minutes with 1000 RPM shaking. Supernatants were collected while hot and cooled to room temperature before Western blot loading. For downstream proteomics analysis, beads were then resuspended in 50 mM Ammonium Bicarbonate buffer containing 1 µg trypsin per sample and incubated at 37 °C overnight. Resulting tryptic peptides were either spin filtered for label-free workflows or carried through for TMT as described below.

TMT Proteomics Procedure and Analysis

The next day, TMT 10plex reagents (Thermo) were equilibrated to room temperature and diluted with 41 µL Optima grade anhydrous acetonitrile. Beads were pelleted and the tryptic-digested supernatant was incubated with the corresponding TMT label: CAR-targeted, 37 °C: TMT 127N, 127C, 128N. CAR-targeted, 4 °C: TMT 128C, 129N, 129C. Control: TMT 130N, 130C, 131. Reaction was incubated at room temperature for 2 hours and then quenched with 8 µL of 5% hydroxylamine for 15 minutes. Samples were then pooled in a new Eppendorf tube and quenched with 12 µL TFA. Samples were stored at –80 °C before being submitted to the Princeton proteomics core.

Mass spectra were obtained using an Orbitrap Fusion at Princeton Proteomics Facility and analysed using MaxQuant. TMT labeled peptides were dried down in SpeedVac, re-dissolved in 300 µl of 0.1% TFA in water and fractionated into 8 fractions using Pierce™ High pH ReversedPhase Peptide Fractionation Kit (#84868). Fractions 1, 4, and 7 were combined as sample 1. Fractions 2 and 6 were combined as sample 2. Fractions 3, 5, and 8 were combined as sample 3. Three combined samples were dried completely in a SpeedVac and resuspended in 20 µl 5% acetonitrile/water (0.1% formic acid (pH = 3)). 2µl (~ 360ng) was injected per run using an EasynLC 1200 UPLC system. Samples were loaded directly onto a 45cm long 75um inner diameter nano capillary column packed with 1.9um C18-AQ resin (Dr. Maisch, Germany) mated to metal emitter in-line with an Orbitrap Fusion Lumos (Thermo Scientific, USA). Column temperature was set at 45 ºC and two-hour gradient method with 300nl per minute flow. The mass spectrometer was operated in data dependent mode with synchronous precursor selection (SPS) - MS3 method (7) with 120,000 resolution of MS1 scan (positive mode, profile data type, Intensity threshold 5.0e3 and mass range of 375-1600 m/z) in the Orbitrap followed by CID fragmentation in ion trap with 35% collision energy for MS2 and HCD fragmentation in Orbitrap (50,000 resolution) with 55% collision energy for MS3. As ratio suppression is a potential concern with TMT-based proteomics, we elected to utilize an MS3 workflow, which can greatly reduce this effect.[4] MS3 scan range was set at 100-500 with injection time of 120ms. Dynamic exclusion list was invoked to exclude previously sequenced peptides for 60s and maximum cycle time of 2.5s was used. Peptides were isolated for fragmentation using quadrupole (0.7 m/z isolation window). Ion-trap was operated in Rapid mode.

Resulting ".raw" data files were converted to ".mzml" format via MSconvert. These files were then processed via MSFragger. TMT-10 Plex MS3 was utilized as the workflow. The human proteome (UP000005640) was utilized as the FASTA, and 'reviewed sequences only', 'add decoys', and 'add common contaminants' were checked. Precursor mass tolerance was set to -20/20 ppm, fragment mass balance tolerance 0.6 Da. Protein digestion settings utilized strict trypsin with a peptide length of 7-50 and a mass range of 200-5000. Modifications included methylation and deamidation, as well as the TMT mass. Upon processing, resulting protein.tsv file was opened in Perseus for processing. Intensity values were converted by transforming by Log2(x), followed by annotating rows to their corresponding experimental condition. For normalization, the median was subtracted, and finally, a volcano plot was generated utilizing the Student's t-test. Resulting volcano plots were plotted in GraphPad Prism 9 for final figures.

Global Proteomics Procedure

For global proteomics profiling, 20 million Jurkat CAR-T cells per replicate (3 biological replicates of the wild-type CAR, 3 biological replicates of the 4-1BB 2× A Mutant) were washed 3× with DPBS. Cells were then resuspended in 500 µL of memPER membrane permeabilization buffer. After 20 minutes of incubation at 4 °C, resulting mixture was centrifuged at 10000×g for 10 minutes to isolate the membrane fraction as a pellet. This pellet was resuspended in 500 µL RIPA containing 1% SDS and cOmplete protease inhibitor. Lysates were then sonicated in the Bioruptor for 10 minutes (high power, water bath 4 °C, 15 seconds on/15 seconds off). Cells were boiled for 10 minutes at 95 °C. After heating, another 600 µL of RIPA with cOmplete was added. Resulting lysates were pelleted at 15000g for 10 minutes, and resulting supernatant transferred to a new tube to clarify lysates. Protein concentrations were measured via BCA assay.

After BCA, 10 µg of total protein for each replicate was cleaned up via the SP3 cleanup protocol.[5] Lysates were first reduced via addition of DTT to each sample to a final concentration of 10 mM for 30 minutes at 55 °C. Following reduction, samples were alkylated via addition of IAA to a final concentration of 15 mM for 30 minutes at room temperature in the dark. To quench IAA, DTT was added to a final concentration of 20 mM to each sample and allowed to incubate 15 minutes at room temperature. After reduction and alkylation, samples were diluted in reconstitution solution, before adding 100 µg total of SP3 beads and homogenizing the solution. The bead-containing

solution was then diluted 1:1 with ethanol and incubated on a Thermo-Mixer at 1000 RPM for 5 minutes at room temperature. Following bead binding, beads were pelleted on a magnetic rack and supernatant was discarded. Beads were washed 3 times with 180 µL 80% ethanol in water solution. Following washes, beads were resuspended in 50 µL of 50 mM ammonium bicarbonate (Sigma-Aldrich, 09830) containing 0.5 µg of MS-grade trypsin (Thermo, 90057) and digested on a Thermo-Mixer at 1000 RPM and 37°C overnight. Following digestion, supernatant was quenched with 0.5 µL Optima-Grade formic acid (Fisher, A117-50). Sample was passed through a 0.22-micron spin filter column (CoStar, 98231-UT-1), before being transferred to an LC-MS vial for proteomics analysis.

Label-Free Proteomics Procedure

Label-free, data-independent analysis (DIA) proteomics was performed on a Bruker TimsTOF Pro 2 in line with a nanoElute LC. Per sample, ~100 ng of protein was first injected onto a trap column (C18 Pepmap, 5 µM particle size, 5 mm length, 300 µM internal diameter), followed by separation on an analytical column (C18 ReproSil AQ, 1.9µM particle size, 100 mm length, 75 µM internal diameter). Peptides were eluted via a gradient consisting of Acetonitrile/Water at a column temperature of 40 °C (buffer A = 0.1% formic acid/water, buffer B = 0.1% formic acid/acetonitrile; flow rate 0.5 µL/min; gradient; start at 2% B, then increase to 35% B over 20 min, increase to 95% B over half a min, hold at 95% for 2.25 min.) Scans were performed in positive ion, dia-PASEF mode over a m/z range of 100-1700 with a ramp time of 100 ms, Accu. time of 100 ms, and a duty cycle of 100%, ramp rate of 9.43 Hz, MS averaging set to 1. Absolute thresholds were set to 5000 for mobility peaks and 10 for MS peaks.

The resulting raw data (.d files) were then processed via DIANN 18.8.1 via the following parameters: trypsin/P digestion, 3 missed cleavages, 3 max. variable modifications, N-term M excision, Ox(M), Ac(N-term) and C-carbamidomethylation, peptide length range of 7-30, precursor charge range 1-4, m/z range 300-1800, fragment ion range 200-1800, Mass accuracy and MS accuracy both set to 10, precursor FDR set to 1%. Within the DIANN algorithm, the following settings are applied: “Use isotopologues”, “MBR” (match between runs), “No shared spectra”, “Heuristic protein inference”. A spectra library was utilized which was generated from DIANN via all known human proteins (In-Silico spectral library – generated in DIANN via FASTA of

Uniprot human proteome UP000005640 – options selected were “FASTA digest for library-free search/library generation” and “Deep learning-based spectra, RTs and IMs prediction’, other parameters same as described above). After processing, resulting matrix.pg files were worked up in Perseus (v. 2.0.7.0). Intensities are inputted as “main” while the other descriptors are listed as “categorical”. Intensities were transformed by log base 2, and data were annotated to the appropriate condition. At this point, normalization was performed via median subtraction, and a volcano plot was generated utilizing a t-test for statistical significance. Resulting volcano plots were plotted in GraphPad Prism 9 for final figures. Interactor overlap was performed with InteractiVenn (https://www.interactivenn.net). Gene ontology analysis was performed via Metascape (https://metascape.org). Heatmaps were created using Morpheus (https://software.broadinstitute.org/morpheus). STRING analysis: StringDB (https://string-db.org). Localization and pathway categorization were manually performed, referring to UniProt (https://www.uniprot.org) and The Human Protein Atlas (https://www.proteinatlas.org).

General Western Blot Protocol

Gel electrophoresis was performed using a Bio–Rad Criterion Vertical Electrophoresis Cell tank, Bio–Rad PowerPac Basic Power Supply, and Criterion TGX tris–glycine polyacrylamide gel cassettes (SDS/Tris). After electrophoresis, gels were transferred from precast cassettes to nitrocellulose or PVDF membranes using an iBlot 2 gel transfer device (Thermo Fisher, IB21001, IB23001), and washed with water. The membranes were then immersed in Odyssey Blocking Buffer (Li–Cor, 927–50000) and incubated for 1 hour. The blocking solution was then decanted to load primary antibody diluted in blocking buffer and incubated overnight at 4 ºC. Membranes were then washed 3× with TBST and incubated with a secondary antibody solution (1:10000) in blocking buffer for at least 1 hour at room temperature. The membranes were washed 3× with TBST and water before imaging via Li–Cor Odyssey CLx scanner in the 700 nm and 800 nm channels.

STED Analysis for Colocalization

CAR-expressing Jurkat cells were cultured and fixed onto poly-L-lysine-coated 24-well glass-bottom plates (Cellvis, 230224) in 4% paraformaldehyde in DPBS. Samples were then washed once with DPBS, permeabilized with 0.1% Triton X-100 in DPBS, and blocked with Blocking buffer (3% BSA, 0.05% Tween20, in DPBS). Primary antibodies diluted in blocking buffer were used for subsequent overnight incubation at 4 °C. Samples were then washed 3× with 0.05% Tw20 in DPBS and stained with secondary antibody solution in Blocking buffer for 1 hour at room temperature. Finally, samples were washed 3× 0.05% Tw20 in DPBS and stored in DPBS before imaging.

Stimulated emission depletion microscopy (STED) was performed on a Nikon Eclipse Ti2 inverted confocal microscope (Nikon, Melville, USA), equipped with a 100x/1.45 oil immersion objective (Nikon plan apo λ), using the STEDYCON software (version 6.0.13339). STED fluorophores were excited with either a 561 or 640 nm laser light from a diode laser (Abberior Instruments, Göttingen, Germany). Lasers were pulsed at a 40 MHz repetition rate with <100 ps pulse width. Stimulated emission depletion was performed with a 775 nm pulser laser (frequency doubled, fiber-amplified diode laser, 40 MHz repetition, 1 ns pulse width). Representative images were analyzed for colocalization in ImageJ/FIJI.

siRNA Knockdown with Nucleofection

HA-CAR Jurkat cells were pelleted and resuspended in 100 μL of complete cell line buffer SE (Lonza, V4XC-1012) per condition. siRNA was added to a final concentration of 1 μM, and the cell suspensions were transferred to 100 μL cuvettes (Lonza, V4XC-1012). Afterwards, the cells were transfected using the Lonza 4D-Nucleofector X Unit (Lonza, AAF-1003X) with the pulse code, CK-116, and were subsequently resuspended in 2 mL of complete media in a 12-well plate. The cells were allowed to recover for 72 hours, after which they were harvested and washed once with PBS. Next, the cells were split into activated and unactivated conditions and activated with anti-HA (abcam, ab9110) in PBS at a 1:1000 concentration for 30 minutes at 37 ºC on a rotator. The cells were washed once, pelleted, and lysed with RIPA supplemented with Halt phosphatase and protease inhibitor cocktail (Thermo, 78440). Concentrations were equalized and lysates were

boiled at 95 ºC with 4x Laemmli (Bio-Rad, 1610747) supplemented with BME. 4-15% gradient gels were run at 150 V for 60 minutes, transferred to nitrocellulose membrane at 100V for 30 minutes, and blocked for 1 hour with 3% BSA in TBST. Membranes were incubated overnight with pZAP70 (CST, 2705S, 1:1000), ZAP70 (CST, 2701S, 1:1000), pERK (CST, 4370S), ERK1/2 (Santa Cruz, sc-514302), PTPRA (Proteintech, 13079-1-AP, 1:2000), TYK2 (Proteintech, 83161-4-RR, 1:5000), or β-actin (CST, 1:1000, 8H10D10). After washing with TBST, membranes were incubated for 1 hour with either goat anti-rabbit (LI-COR, 926-3221, 1:20000) or goat anti-mouse (LI-COR, 926-68070, 1:20000) secondary antibodies and imaged with a LI-COR Odyssey CLx Imager. siRNA product (Dharmacon) used: Scramble: D-001810-10-50; PTPRA: L-004519-00-0005; TYK2: L-003182-00-0005.

Proteomics Principal Component Analysis (PCA)

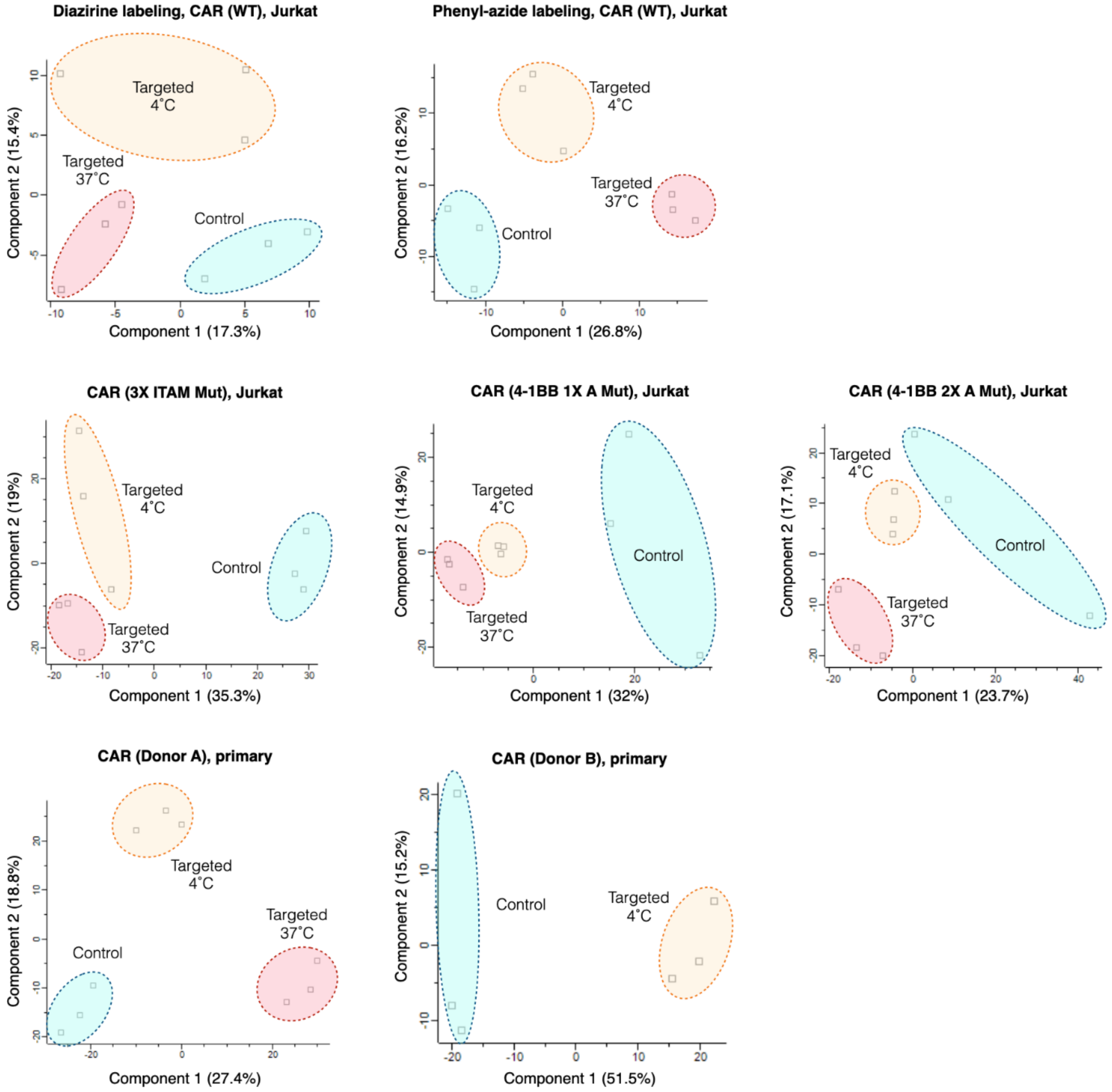

STED Microscopy Uncropped Images

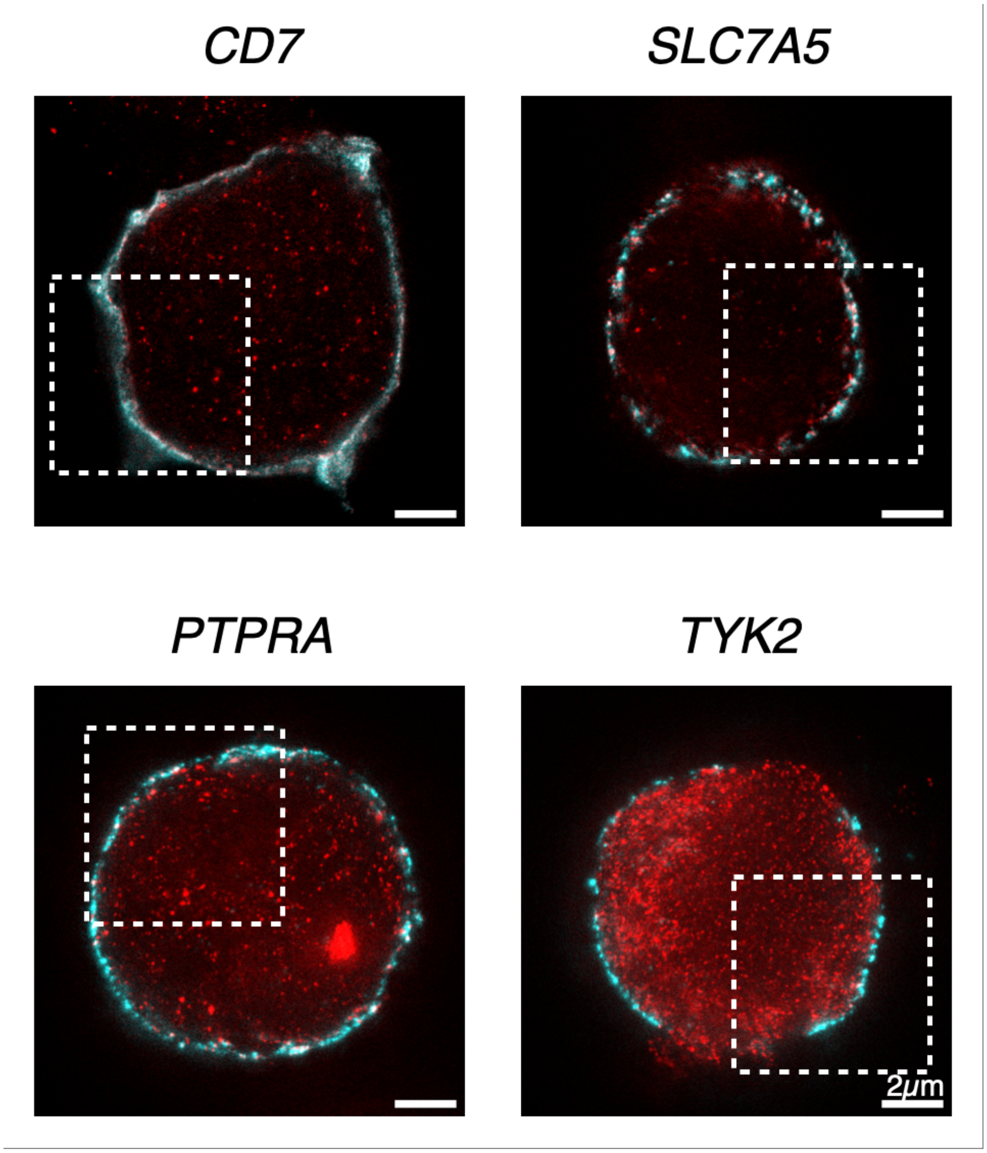

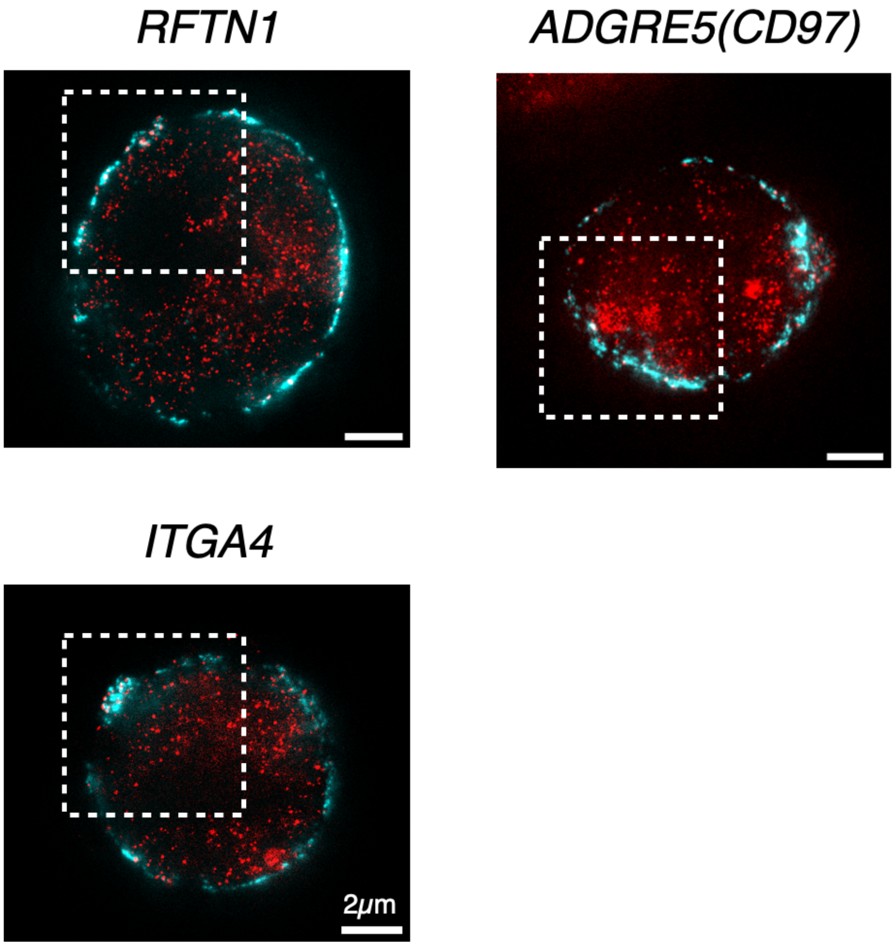
RFTN1
ADGRE5(CD97)
ITGA4
2μm

CAR Sequence

MLLLVTSLLLCELPHPAFLLIPYPYDVPDYADIQMTQTTSSLSASLGDRVTISCRASQDISK
YLNWYQQKPDGTVKLLIYHTSRLHSGVPSRFSGSGSGTDYSLTISNLEQEDIATYFCQQG
NTLPYTFGGGTKLEITGSTSGSGKPGSGEGSTKGEVKLQESGPGLVAPSQSLSVTCTVSG
VSLPDYGVSWIRQPPRKGLEWLGVIWGSETTYYNSALKSRLTIIKDNSKSQVFLKMNSL
QTDDTAIYYCAKHYYYGGSYAMDYWGQGTSVTVSSESKYGPPCPPCPMFWVLVVVGG
VLACYSLLVTVAFIIFWVKRGRKKLLYIFKQPFMRPVQTT**QE**EDGCSCRFPE**EE**EGGCEL
RVKFSRSADAPAYQQGQNQL**Y**NELNLGRREE**Y**DVLDKRRGRDPEMGGKPRRKNPQEG
L**Y**NELQKDKMAEA**Y**SEIGMKGERRRGKGHDGL**Y**QGLSTATKDT**Y**DALHMQALPPR

Signal Peptide / HA Tag / FMC63 Variable Light Chain / Linker / FMC63 Variable Heavy Chain /

IgG4 Hinge / CD28TM / 4-1BB / CD3z

4-1BB mutations:
1×: Tx**Q**E to Tx**A**E and Px**E**E to Px**A**E

2×: Tx**QE** to Tx**AA** and Px**EE** to Px**AA**

CD3z ITAM mutation:
YxxLx$_{(6\text{-}8)}$**Y**xxL --- **Y**xxLx$_{(6\text{-}8)}$**Y**xxI --- **Y**xxLx$_{(6\text{-}8)}$**Y**xxL to
FxxLx$_{(6\text{-}8)}$**F**xxL --- **F**xxLx$_{(6\text{-}8)}$**F**xxI --- **F**xxLx$_{(6\text{-}8)}$**F**xxL

Donor Information

Category	Donor A (PAD-20099)	Donor B (PAD-20133)
**Sex**	Male	Male
**Age**	26	44
**Ethnicity**	African-American	African-American
**Blood Type (ABO/Rh)**	O+	A+
**Health Status**	Normal healthy subject	Normal healthy subject
**Collection Method**	Leukapheresis (non-mobilized MNC)	Leukapheresis (non-mobilized MNC)
**Target Blood Volume**	~12 L	~12 L
**MNC Product Volume**	203 mL	202 mL
**Total Volume (incl. plasma)**	353 mL	352 mL
**Shipping Conditions**	2–8 °C	2–8 °C
**Collection Date**	Feb 13, 2020	Feb 20, 2020
**WBC (product)**	~104 $\times 10^3/\mu L$	~67 $\times 10^3/\mu L$
**Notes**	Standard QC, screened for infectious diseases	Standard QC, screened for infectious diseases

References

(1) Geri, J. B.; Oakley, J. V.; Reyes-Robles, T.; Wang, T.; McCarver, S. J.; White, C. H.; Rodriguez-Rivera, F. P.; Parker, D. L.; Hett, E. C.; Fadeyi, O. O.; Oslund, R. C.; MacMillan, D. W. C. Microenvironment Mapping via Dexter Energy Transfer on Immune Cells. *Science* **2020**, *367* (6482), 1091–1097. https://doi.org/10.1126/science.aay4106.

(2) Seath, C. P.; Burton, A. J.; Sun, X.; Lee, G.; Kleiner, R. E.; MacMillan, D. W. C.; Muir, T. W. Tracking Chromatin State Changes Using Nanoscale Photo-Proximity Labelling. *Nature* **2023**, *616* (7957), 574–580. https://doi.org/10.1038/s41586-023-05914-y.

(3) Suzuki, S.; Geri, J. B.; Knutson, S. D.; Bell-Temin, H.; Tamura, T.; Fernández, D. F.; Lovett, G. H.; Till, N. A.; Heller, B. L.; Guo, J.; MacMillan, D. W. C.; Ploss, A. Photochemical Identification of Auxiliary Severe Acute Respiratory Syndrome Coronavirus 2 Host Entry Factors Using μMap. *J. Am. Chem. Soc.* **2022**, *144* (36), 16604–16611. https://doi.org/10.1021/jacs.2c06806.

(4) Ting, L.; Rad, R.; Gygi, S. P.; Haas, W. MS3 Eliminates Ratio Distortion in Isobaric Multiplexed Quantitative Proteomics. *Nature Methods* **2011**, *8* (11), 937–940. https://doi.org/10.1038/nmeth.1714.

(5) Hughes, C. S.; Moggridge, S.; Müller, T.; Sorensen, P. H.; Morin, G. B.; Krijgsveld, J. Single-Pot, Solid-Phase-Enhanced Sample Preparation for Proteomics Experiments. *Nature Protocols* **2019**, *14* (1), 68–85. https://doi.org/10.1038/s41596-018-0082-x.
